# Branched-chain amino acids govern the high learning ability phenotype in Tokai high avoider (THA) rats

**DOI:** 10.1038/s41598-021-02591-7

**Published:** 2021-11-29

**Authors:** Yukari Shida, Hitoshi Endo, Satoshi Owada, Yutaka Inagaki, Hideaki Sumiyoshi, Akihide Kamiya, Tomoo Eto, Masayuki Tatemichi

**Affiliations:** 1 Center for Molecular Prevention and Environmental Medicine, Department of Preventive Medicine, Tokai University School of Medicine, 143 Shimokasuya, Isehara, Kanagawa 259-1193 Japan; 2 Center for Matrix Biology and Medicine, Department of Innovative Medical Science, Tokai University School of Medicine, 143 Shimokasuya, Isehara, Kanagawa 259-1193 Japan; 3 Department of Molecular Life Sciences, Tokai University School of Medicine, 143 Shimokasuya, Isehara, Kanagawa 259-1193 Japan; 4 Central Institute for Experimental Animals, 3-25-12 Tonomachi, Kawasaki-ku, Kawasaki, Kanagawa 210-0821 Japan

**Keywords:** Animal behaviour, Metabolomics, Operant learning, Behavioural methods, Psychophysics

## Abstract

To fully understand the mechanisms governing learning and memory, animal models with minor interindividual variability and higher cognitive function are required. THA rats established by crossing those with high learning capacity exhibit excellent learning and memory abilities, but the factors underlying their phenotype are completely unknown. In the current study, we compare the hippocampi of parental strain Wistar rats to those of THA rats via metabolomic analysis in order to identify molecules specific to the THA rat hippocampus. Higher branched-chain amino acid (BCAA) levels and enhanced activation of BCAA metabolism-associated enzymes were observed in THA rats, suggesting that acetyl-CoA and acetylcholine are synthesized through BCAA catabolism. THA rats maintained high blood BCAA levels via uptake of BCAAs in the small intestine and suppression of BCAA catabolism in the liver. Feeding THA rats with a BCAA-reduced diet decreased acetylcholine levels and learning ability, thus, maintaining high BCAA levels while their proper metabolism in the hippocampus is the mechanisms underlying the high learning ability in THA rats. Identifying appropriate BCAA nutritional supplements and activation methods may thus hold potential for the prevention and amelioration of higher brain dysfunction, including learning disabilities and dementia.

## Introduction

Behavioral studies utilize experimental rodent models to explore brain function, including learning and memory processes^1,2^. Rodent models are of particular relevance for investigating the molecular pathophysiology of and developing treatments for higher brain dysfunction conditions such as Alzheimer’s and Parkinson’s diseases, traumatic brain injury, posttraumatic stress disorder, and other neurological conditions^1^. Rodents are suitable for performing behavioral tests to evaluate cognitive ability and thus, are indispensable experimental models in the field of behavioral science. As the molecular mechanisms involved in learning and memory formation in the brain share considerable similarities between humans and rodents^3^, novel approaches for the prevention and treatment of learning deficits can be developed through studying animal models of excellent learning ability.

We previously established and maintained Tokai high avoider (THA) rats, a unique rat strain without any genetic manipulation, which is derived from JCL-Wistar rats^4–6^. THA rats were generated via the selective mating of sibling rats that exhibited a high avoidance rate in the free-operant behavioral task based on the Sidman avoidance schedule. THA rats are unique experimental animals with a connaturally guaranteed high learning ability, strong memory, rapid acquisition of behavioral skills, and minor interindividual differences^4–6^. Owing to their abilities to learn and remember by quickly adapting to previously inexperienced conditions and environments, THA rats have exceptional results in various behavioral tests when compared to Wistar rats^4–9^. Since the THA rat strain was established through repeated mating for more than 100 generations to date^10,11^, we speculate that specific molecular factors are underlying the rats’ high learning ability. Therefore, we sought to identify molecules that promote learning and memory by studying the THA phenotype.

The metabolome is defined as the complete set of metabolites implicated in the interaction between gene expression, protein expression, and the cellular environment^12,13^. Metabolome analysis entails the comprehensive measurement of all metabolites in a biological specimen, including amino acids, monosaccharides, small lipids, cofactors, vitamins, energy cycle intermediates, nucleotides, and xenobiotics^12,13^. The number of metabolites profiled via metabolomics is much larger than through the assessment of known biomarkers and biochemical indexes, allowing for more comprehensive coverage of biological processes and metabolic pathways^14–16^. As metabolites are closely related to phenotype, and the metabolome is sensitive to various factors affecting the phenotype^12,13,16^, different metabolites are expected to characterize different learning states. Therefore, brain metabolome analysis of animal strains with different learning abilities would allow for the large-scale quantitative profiling of metabolites that are essential for cognitive function, offering a major avenue for advances in neurobehavioral science^17^. The above-described utility of metabolome analysis prompted us to identify the distinctive THA rat metabolites and their roles in sustaining the observed learning and memory phenotype. Therefore, the purpose of this study is to identify factors involved in the phenotype of high learning ability in THA rats. Herein, we report that branched-chain amino acids (BCAAs), including leucine (Leu), isoleucine (Ile), and valine (Val) are essential determinants of high learning ability in THA rats.

## Results

### High levels of BCAAs are present in the hippocampus of THA rats

**Figure 1:**
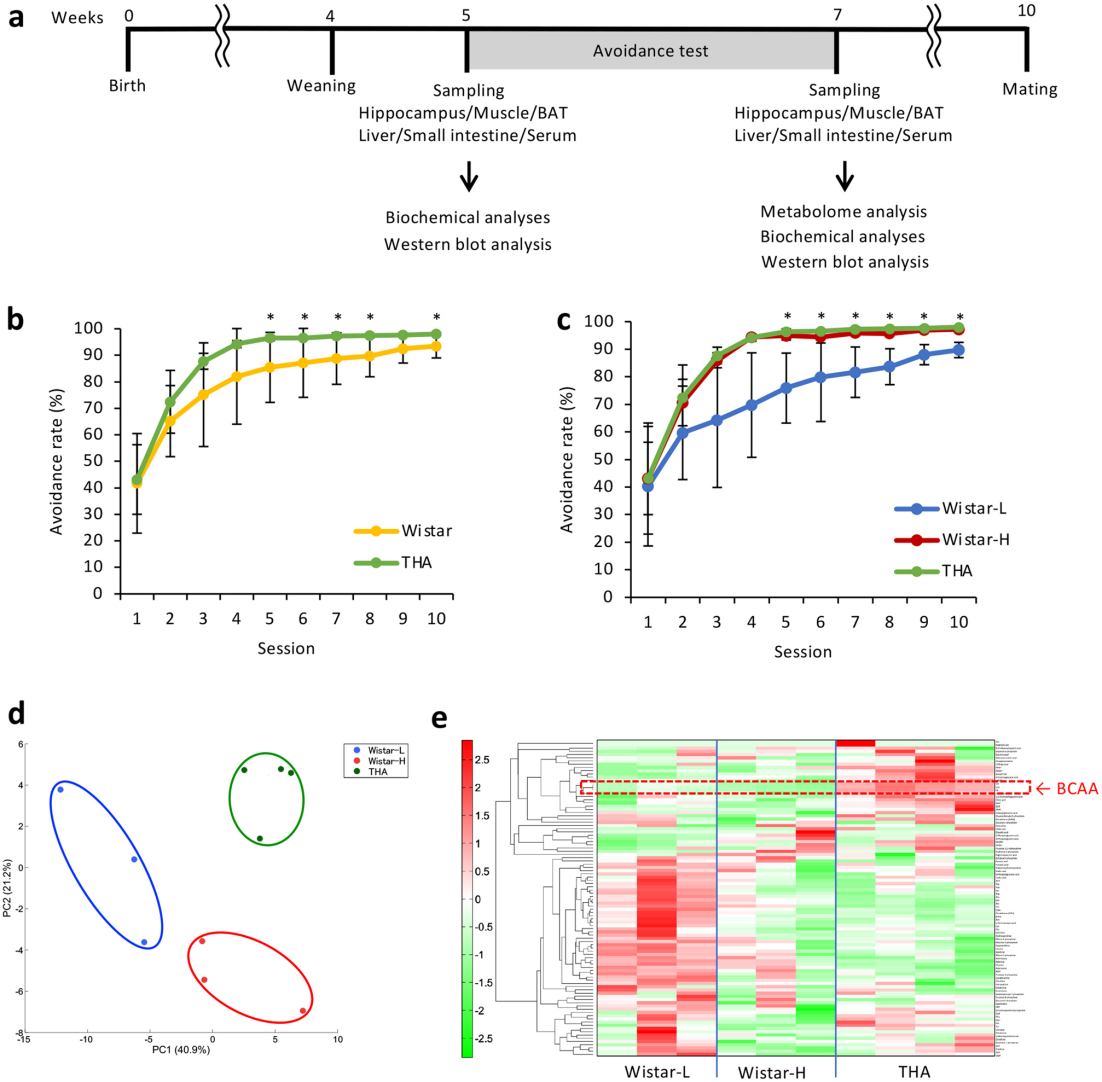
Determination of metabolites involved in THA rat-specific high learning ability via metabolome analysis. (**a**) Schematic representation of the experimental protocol. The animals used in the experiment were bred in the same breeding environment. After weaning at 4 weeks of age, a behavioral avoidance test was conducted at 5 weeks of age. Sampling and experiments were carried out at the times described. (**b**) Comparison of avoidance rate during the lever-pressing test between Wistar (n = 6) and THA rats (n = 4). (**c**) Avoidance rates of THA rats, Wistar rats (Wistar-H, n = 3) showing high avoidance rate, and other Wistar rats (Wistar-L, n = 3). Data are expressed as the mean ± SD values. *P < 0.05. (**d**) Principal component analysis (PCA) of results obtained from metabolome analysis of the hippocampus in Wistar-L, Wistar-H, and THA rats. PCA was conducted using SampleStat software. Blue enclosure; Wistar-L, red enclosure; Wistar-H, and green enclosure; THA rats. (**e**) Hierarchical clustering analysis (HCA) of the metabolites in Wistar-L, Wistar-H, and THA rats. HCA was conducted using PeakStat software. Red indicates an increase of metabolites, and green indicates a decrease, respectively. The part surrounded by the red dotted line indicates BCAAs.

To examine differences in learning ability, we performed a free-operant behavioral avoidance test following the Sidman schedule in both Wistar and THA rats at 5 weeks of age. Consistent with previous reports^10^, THA rats had a high avoidance rate and very small interindividual variability, immediately learning the components of the test (Fig. 1b). In contrast, Wistar rats exhibited a gradual increase in avoidance rate as the number of trials increased, and there was not much improvement in the considerable individual variability in avoidance rate. Interestingly, the avoidance rate of all Wistar rats could be divided into two groups, namely those with similar high-performance levels in the test, as observed for THA rats (abbreviated here as Wistar-H), and the rest (shown as Wistar-L) (Fig. 1c). To identify the factors associated with the observed THA phenotype, we performed metabolome analysis using the hippocampi from Wistar-L, Wistar-H, and THA rats obtained after the test. Principal component analysis (PCA) of metabolites indicated that the score plot of each of the three groups was apparent and independently clustered (Fig. 1d). Hierarchical cluster analysis (HCA) suggested that only six metabolites, including BCAAs, were significantly higher in the hippocampus of THA rats compared to both Wistar-L and Wistar-H rats, whereas alanine (Ala) and adenosine were significantly lower (Fig. 1e, Table 1). The high levels of BCAAs in the hippocampus of THA rats were particularly apparent during the comparison of essential amino acids (Supplementary Fig. S2 online). These results suggest that high BCAA levels in the hippocampus are specific to THA rats and thus may be major determinants of high learning ability.

**Table 1:** Significantly higher or lower metabolites in THA rats and their corresponding classifications in the KEGG and HMDB.

Compound name	KEGG ID	HMDB ID	Comparative analysis			
			THA vs Wistar-L		THA vs Wistar-H	
			Ratio	*p*-value	Ratio	*p*-value
**Higher metabolites**						
Val	C00183	HMDB00883	2.5	0.002	5.6	5.8E−05
Ile	C00407	HMDB00172	2.9	4.1E−04	5.1	1.2E−04
Leu	C00123	HMDB00687	2.1	7.8E−04	3.4	2.8E-04
cAMP	C00575	HMDB00058	1.2	0.039	1.3	0.010
NADP+	C00006	HMDB00217	1.4	0.015	1.5	0.017
Citric acid	C00158	HMDB00094	1.2	0.042	1.3	0.039
**Lower metabolites**						
Ala	C00041	HMDB00161	0.6	0.014	0.8	0.008
Adenosine	C00212	HMDB00050	0.5	0.002	0.6	0.006

KEGG, Kyoto Encyclopedia of Genes and Genomes; HMDB, Human Metabolome Database.

### Activation of BCAA metabolism and the acetylcholine synthesis pathway in the hippocampus of THA rats supports high learning ability

**Figure 2:**
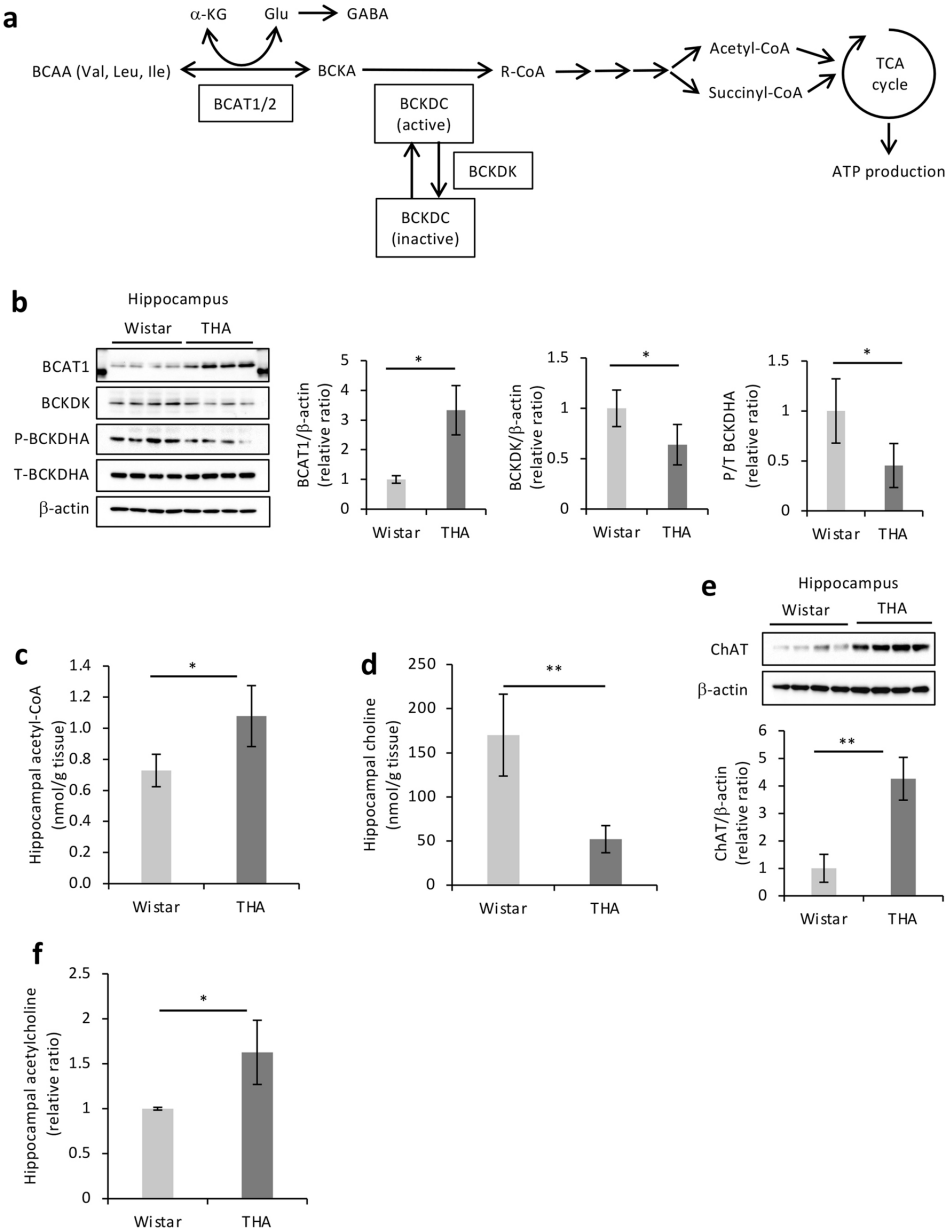

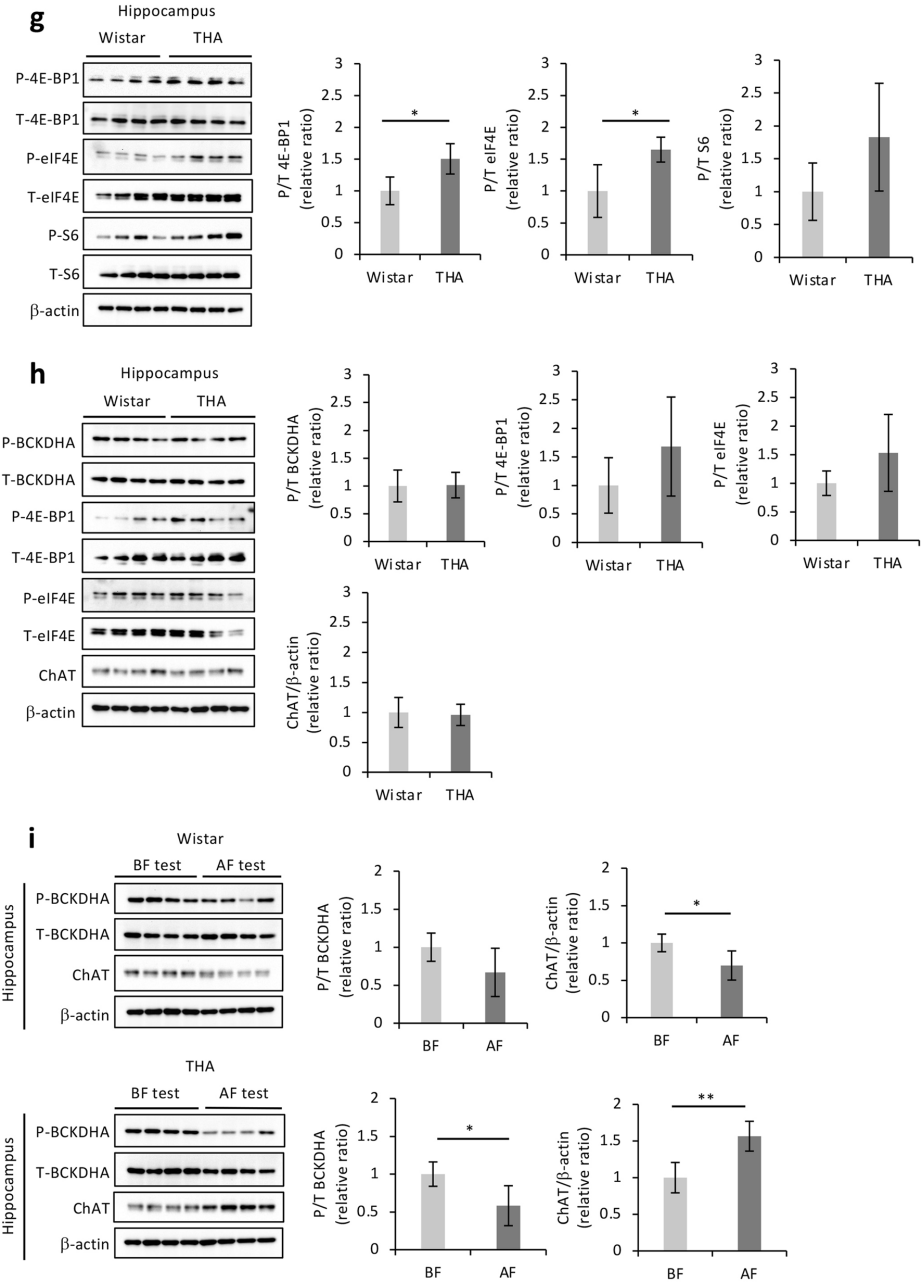
BCAAs are oxidized in the hippocampus of THA rats in response to the learning test. (**a**) Schematic presentation of the BCAA oxidation pathway. BCAAs are transaminated by BCAT1 or BCAT2 to generate BCKAs, which are subsequently oxidized by BCKDC. α-KG is the α-keto acid acceptor of the BCAA nitrogen group, and Glu is the product, which is utilized as a substrate for GABA synthesis. BCKDC is phosphorylated and inactivated by BCKDK. BCAA-derived intermediates are trapped in the mitochondria by CoA. The end products of the BCAA catabolic pathway, succinyl CoA and acetyl CoA act as TCA cycle intermediates for ATP production. R-CoA: acyl CoA. (**b**) Western blot analyses of BCAT1, BCKDK, phosphorylated BCKDHA (P-BCKDHA), and total BCKDHA (T-BCKDHA) as well as their quantified ratios in the hippocampi of Wistar rats (n = 4) and THA rats (n = 4) after avoidance tests. β-actin served as a loading control. (**c**) Acetyl-CoA and (**d**) choline concentrations in the hippocampus after the avoidance test (Wistar rats n = 6, THA rats n = 4). (**e**) ChAT expression in protein extracts from the hippocampus. β-actin served as a loading control. The expression level was determined via densitometric analysis. (**f**) Hippocampal acetylcholine levels in the above tissues (Wistar rats n = 6, THA rats n = 4). (**g**) Western blot analyses of phosphorylated 4E-BP1 (P-4E-BP1), total 4E-BP1 (T-4E-BP1), phosphorylated eIF4E (P-eIF4E), total eIF4E (T-eIF4E), phosphorylated S6 (P-S6), and total S6 (T-S6) expression in the above tissues. β-actin served as a loading control. The expression level was determined via densitometric analysis. (**h**) Western blot analyses of P-BCKDHA, T-BCKDHA, P-4E-BP1, T-4E-BP1, P-eIF4E, T-eIF4E, and ChAT and their quantified ratios in the hippocampi of Wistar rats and THA rats before the avoidance test. (**i**) Comparison of P-BCKDHA, T-BCKDHA, and ChAT expression before (BF) and after (AF) avoidance tests via western blot analyses of hippocampi from Wister (n = 4) or THA rats (n = 4), respectively. β-actin served as a loading control. The expression level was determined via densitometric analysis. Data are expressed as the mean ± SD values. *P < 0.05; **P < 0.01. Uncropped blots are presented in Supplementary Fig. S6.

Most of the energy required to support neuronal function is generated via glycolysis and mitochondrial oxidative phosphorylation^18^. Unexpectedly, no significant differences in glucose metabolism within the hippocampus were observed between any of the groups (Supplementary Fig. S3A online). ATP levels also were not significantly different among the three groups (Supplementary Fig. S3B online), suggesting that the THA rat-specific learning ability is regulated by the activation of other metabolic pathways in addition to glucose-dependent or -independent energy metabolism. The activation of BCAA catabolism is known to be a source of energy within the central nervous system (CNS)^19,20^. We, therefore, speculated that BCAA metabolism in the hippocampus plays an important role in determining the learning ability of THA rats. BCAAs are metabolized through reversible transamination reactions by BCAT proteins, consecutively catalyzed by the branched-chain α-keto acid dehydrogenase (BCKDH) enzyme complex through an irreversible oxidative decarboxylation step^21^. The metabolic activity of BCKDH is determined by the phosphorylation state of its E1 subunits regulated by the BCKDK phosphatase. The phosphorylated form renders the complex inactive, thereby giving rise to acetyl-coenzyme A (acetyl-CoA) and succinyl-CoA content, resulting in the utilization of TCA-generated ATP as an energy source (Fig. 2a). To investigate whether the activation of BCAA metabolism is involved in the high learning ability of THA rats, the expression of BCAA metabolic enzymes in hippocampi obtained after the learning test was examined. Since our results suggested that high BCAA levels play a central role in THA rat learning ability (Table 1, Supplementary Fig. S2 online), we compared THA rats with Wistar rats, which had no other phenotypic differences. As expected, while the expression of BCAT1, a peripheral nervous system-specific isoform^22,23^, was increased, that of BCKDK and the phosphorylation levels of BCKDHA were lower in the hippocampi of THA rats compared to Wistar rats (Fig. 2b). In addition, acetyl-CoA was significantly higher in THA rats (Fig. 2c), suggestive of BCAA metabolism activation in the hippocampus after the learning test. The neurotransmitter acetylcholine is synthesized by choline acetyltransferase (ChAT) using both acetyl-CoA and choline as substrates^24^. Choline levels were low in THA rats (Fig. 2d). Consistent with these results, the expression level of ChAT in the hippocampus of THA rats was markedly high and inversely correlated with choline depletion (Fig. 2e). As expected, the acetylcholine levels were also significantly higher (Fig. 2f). Although BCAAs can be utilized as substrates for synthesizing glutamate and γ-aminobutyric acid (GABA) (Fig. 2a)^19,20^, there was no difference in respective metabolite levels among Wistar-L, Wistar-H, and THA rats (Supplementary Fig. S3C,D online). We speculated that BCAA catabolism and the activation of the acetylcholine synthesis contribute to the improvement of learning and memory abilities. Notable phosphorylation of eukaryotic translation initiation factor 4E (eIF4E) and eIF4E-binding protein 1 (4E-BP1), but not of ribosomal protein S6 (S6), all of which are markers of long-term memory^25^, was also detected in the hippocampus of THA rats, as compared with Wistar rats after the behavioral test (Fig. 2g). No suppression of BCKDHA phosphorylation, enhanced phosphorylation of 4E-BP1 and eIF4E, as well as increased expression of ChAT were observed in the hippocampus of THA rats prior to the learning test (Fig. 2h). Furthermore, an apparent suppression of BCKDHA phosphorylation and upregulation of ChAT were observed only in the hippocampus of THA rats as changes before and after the learning test (Fig. 2i). In contrast, the hippocampal expression of ChAT in Wistar rats decreased after the learning test (Fig. 2i), suggestive of difficulty in improving the desired learning behavioral performance. The results obtained from multilevel comparisons clearly indicated that THA rats could achieve high avoidance ability through improved learning and memory retention capacity promoted by BCAA metabolism in the hippocampus.

### High levels of serum BCAAs in THA rats are maintained through their enhanced absorption via the small intestine and reduced catabolism in the liver

**Figure 3:**
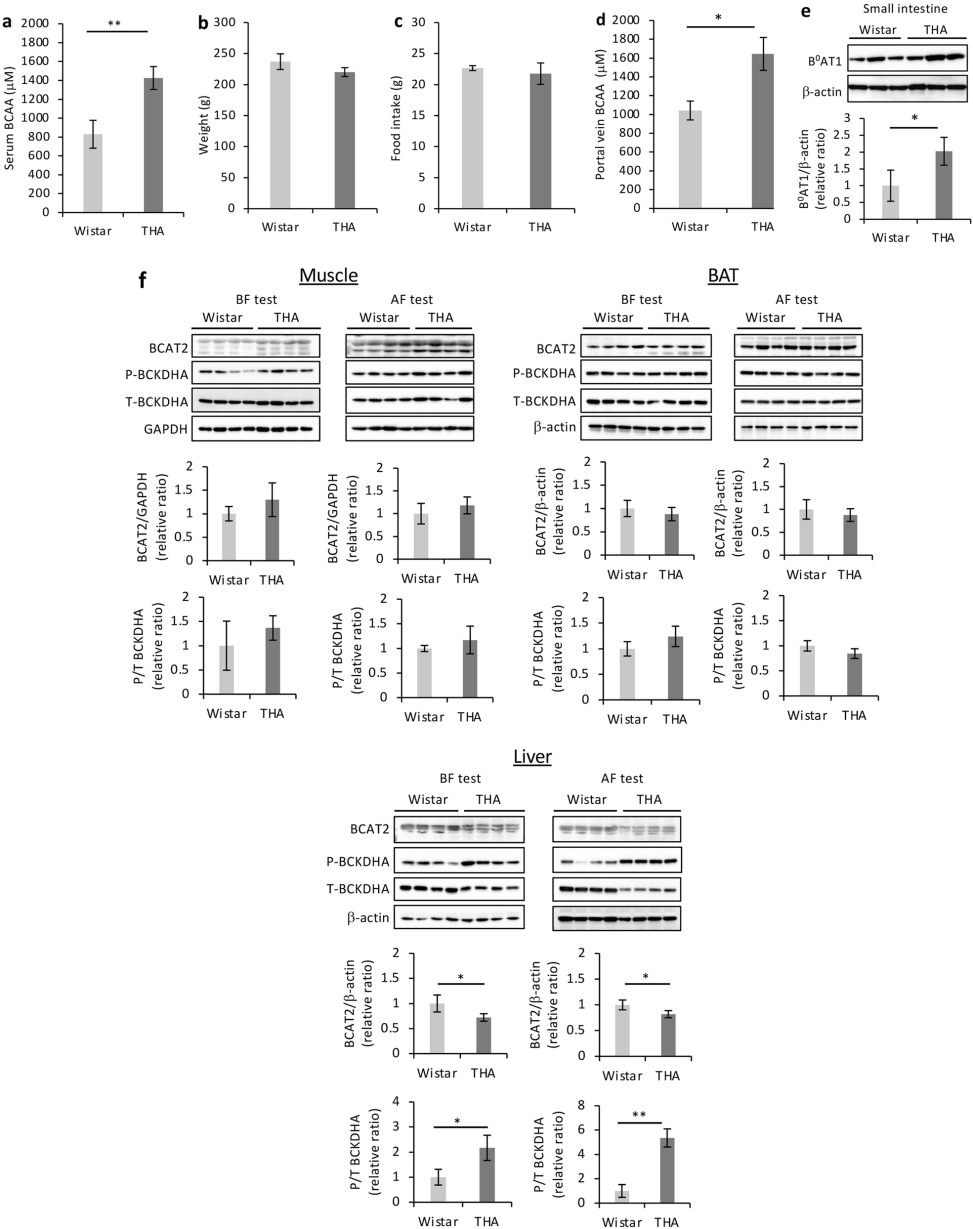
Mechanism of blood BCAA maintenance by molecules involved in absorption and oxidation. Mechanism of blood BCAA maintenance by molecules involved in absorption and oxidation. (**a**) Serum BCAA concentration in Wistar (n = 4) and THA (n = 4) rats after avoidance tests. (**b**) Bodyweight (Wistar rats n = 4, THA rats n = 5), (**c**) dietary intake during 24-h period (Wistar rats n = 4, THA rats n = 5), (**d**) BCAA concentration in the hepatic portal vein, and (**e**) western blot analysis of small intestine tissue probed with antibodies against B^0^AT1 with β-actin as a loading control (Wistar rats n = 3, THA rats n = 3). A relative ratio comparison is shown. (**f**) Comparison of indicated tissues between Wistar (n = 4) and THA (n = 4) rats before (BF test) or after (AF test) the avoidance test. Western blot analyses of BCAT2, P-BCKDHA, and T-BCKDHA and their quantified ratios. GAPDH or β-actin was used as a loading control. Data are expressed as the mean ± SD values. *P < 0.05; **P < 0.01. Uncropped blots are presented in Supplementary Fig. S6.

Next, we investigated the mechanism through which the high levels of BCAAs are maintained in the hippocampus of THA rats. The BCAA concentration in serum obtained from the peripheral veins of THA rats was higher than that of Wistar rats (Fig. 3a). Meanwhile, there was no significant difference in body weight and dietary intake between the two groups after the learning test (Fig. 3b,c). BCAAs belong to the group of essential amino acids which in animals are most often obtained from dietary sources rather than endogenous synthesis^26^, suggesting that the absorption of dietary BCAAs in the small intestine was enhanced in THA rats compared to Wistar rats. Indeed, portal vein-derived serum BCAA levels were significantly higher in THA rats than in Wistar rats (Fig. 3d). B^0^AT1 is the major transporter of neutral amino acids in the intestinal epithelia, transferring the bulk of diet-derived BCAAs from the apical side^27,28^. While BCAT2 expression and BCKDHA activation tended to increase within the small intestine of THA rats (Supplementary Fig. S4A online), B^0^AT1 expression was significantly higher than in Wistar rats after the learning behavioral test (Fig. 3e). Similar results were obtained for the comparison prior to the learning test (Supplementary Fig. S4B online). BCAA levels in the portal vein and serum were also significantly higher in THA rats than in Wistar rats (Supplementary Fig. S4C,D online). These results suggested that THA rats have higher blood BCAA levels than Wistar rats due to a superior absorption of BCAAs from the small intestine. When compared with the livers of Wistar rats, those of THA rats showed suppression of BCAT2 expression and increased phosphorylation of BCKDHA before and after the avoidance learning test, while no such differences were observed in muscle and brown adipose tissue (BAT) (Fig. 3f). The decreased expression of BCAT2 and increased phosphorylation of BCKDHA were caused by the behavioral avoidance test, and this phenomenon was observed only in the livers of THA rats, but not in those of Wistar rats (Supplementary Fig. S4E online). Considered together, these results indicated that THA rats consistently maintained high BCAA blood levels by increasing BCAA absorption from the small intestine and suppressing BCAA catabolism in the liver in preparation for the high demand for BCAA metabolism in the hippocampus during behavioral avoidance tests.

### BCAAs play a vital role in the general development and maintenance of high learning ability in THA rats

**Figure 4:**
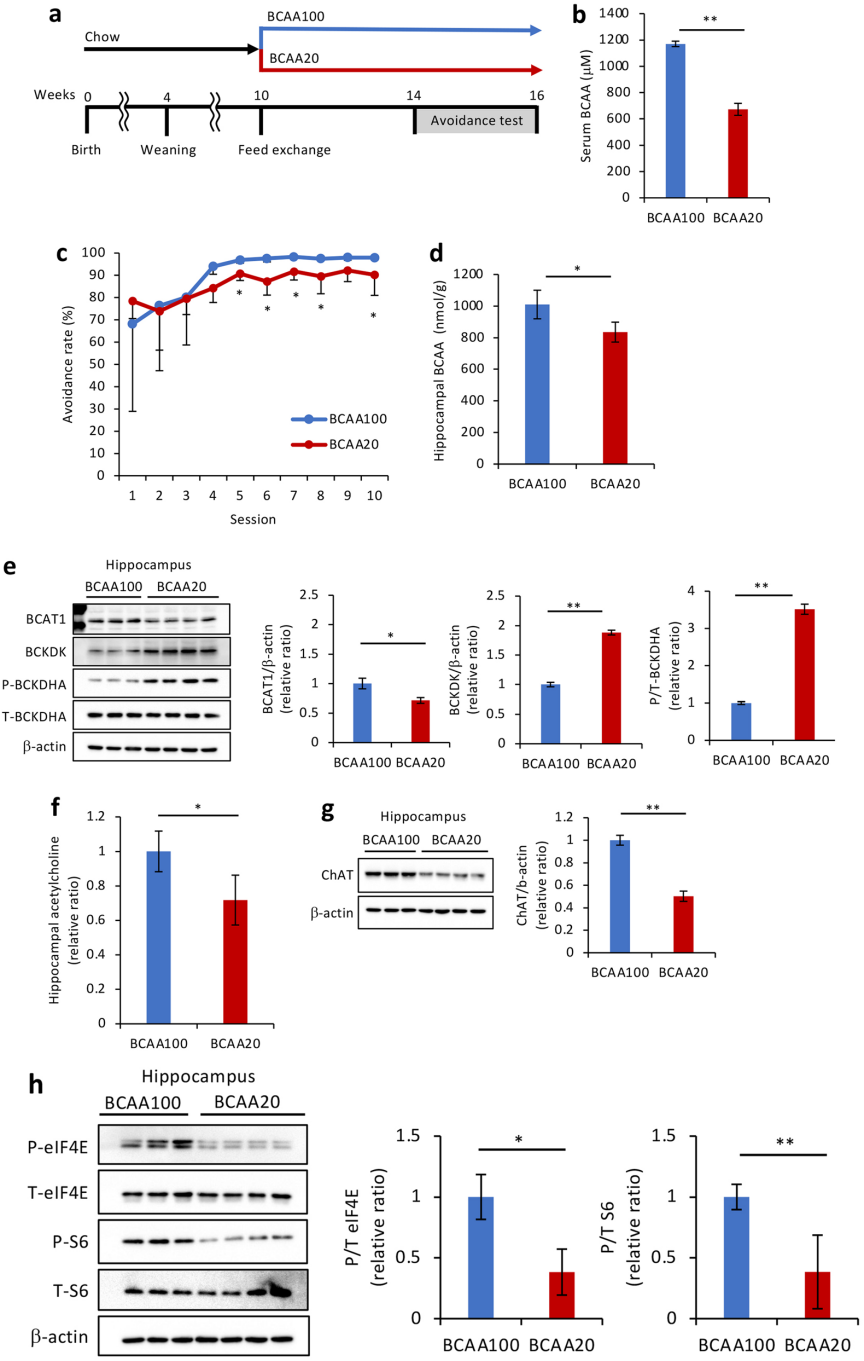
Effects of BCAA-restricted diet on learning ability and metabolism. (**a**) Schematic representation of the experiments with a BCAA-reduced diet. BCAA100: control (n = 6), BCAA20: one-fifth BCAA content of the control (n = 8). (**b**) Serum BCAA concentration in BCAA100 and BCAA20 groups after avoidance tests. (**c**) Comparison of the avoidance rate during the lever-pressing avoidance behavior test in BCAA100 and BCAA20 groups. (**d**) Hippocampal BCAA concentration in BCAA100 and BCAA20 groups after avoidance tests. (**e**) Western blot analyses of BCAT1, BCKDK, P-BCKDHA, and T-BCKDHA expression levels and their quantified ratios in the above tissues with β-actin as a loading control. (**f**) Hippocampal acetylcholine levels in the above tissues. Western blot analyses of (**g**) ChAT, (**h**) P-eIF4E, T-eIF4E, P-S6, and T-S6 and their quantified ratios in the above tissues with β-actin as a loading control. Data are expressed as the mean ± SD values. *P < 0.05; **P < 0.01. Uncropped blots are presented in Supplementary Fig. S6.

To determine in greater detail whether BCAA affects learning ability, we fed THA rats with BCAA-reduced diets and corresponding control diets. Diet protein content was modified so that the BCAA20 diet contained one-fifth of the BCAA content of the control diet BCAA100 (AIN93-G), which has a standard amount of BCAAs (Table 2). Corn starch was added to compensate for the reduction in energy content due to BCAA reduction in the diet. We fed rats the BCAA-reduced diet at 4 weeks of age, subsequently initiated the behavioral avoidance test at 5 weeks of age (Supplementary Fig. S5A online). Although serum BCAA levels were lower in rats fed the 3-week BCAA-reduced diet (Supplementary Fig. S5B online), we unexpectedly observed that BCAA20-fed rats exhibited no increase in weight gain or dietary intake immediately after the intervention relative to the control BCAA100-fed group, indicative of a suppressed healthy growth (Supplementary Fig. S5C,D online). Therefore, we concluded that the effect of BCAA reduction on learning behavior could not be accurately evaluated. To avoid this growth inhibition, we evaluated the effects of BCAA-reduced dietary interventions in nearly mature 10-week-old rats (Fig. 4a). In the preliminary analyses, we confirmed that there was almost no difference in learning performance between 5 and 10 weeks of age (Supplementary Fig. S5E online), and the phosphorylation of BCKDHA within the hippocampus was decreased by the learning test at 10 weeks of age (Supplementary Fig. S5F online). These results indicate that the metabolic regulation of BCAAs in the hippocampus plays an important role in learning and memory in THA rats, regardless of age. Similar to a previous report of a protein-restricted diet^29^, no reduction in food intake and marked growth inhibition were observed in the BCAA20-fed group from 10 weeks of age (Supplementary Fig. S5G,H online). After confirming that serum BCAA concentrations were sufficiently reduced to almost the same as those in Wistar rats by feeding THA rats with BCAA20 for 4 weeks (Fig. 4b), a behavioral avoidance test was performed. As expected, avoidance rates in the BCAA20-administered group showed a downward trend throughout, exhibiting a significant decrease from the 5th to 8th as well as in the 10th session (Fig. 4c). The amount of BCAAs in the hippocampus was sufficiently reduced due to BCAA20 feeding (Fig. 4d), decreasing the expression of BCAT1 and drastically upregulating BCKDK expression as well as BCKDHA phosphorylation (Fig. 4e). Notably, feeding BCAA20 caused an obvious decrease in the levels of acetylcholine in addition to the suppression of ChAT expression in the hippocampus (Fig. 4f,g). Phosphorylation of eIF4E and S6 was also significantly diminished, supporting the learning ability decline induced by BCAA-reduced diets (Fig. 4h). Considered together, our observations strongly suggest that BCAAs are essential nutrients for the high learning ability of THA rats, with BCAA blood levels and BCAA metabolism activation in the hippocampus playing a central role in the maintenance of this cognitive phenotype.

**Table 2:** Nutrient composition of the BCAA100 and 20 diets.

Product #	BCAA100	BCAA20
	A10012G	A19122301
**Amino acid (g/kg)**		
l-alanine	5.08	5.08
l-arginine	6.00	6.00
l-asparagine, monohydrate	7.12	7.12
l-aspartate	5.08	5.08
l-cysteine	4.27	4.27
l-glutamate	21.05	21.05
l-glutamine	17.39	17.39
l-glycine	3.05	3.05
l-histidine	4.58	4.58
l-lysine	13.32	13.32
l-methionine	5.08	5.08
l-phenylalanine	8.54	8.54
l-proline	17.90	17.90
l-serine	10.07	10.07
l-threonine	7.22	7.22
l-tryptophan	2.14	2.14
l-tyrosine	9.25	9.25
l-leucine	15.96	3.15
l-isoleucine	7.63	1.53
l-valine	9.35	1.93
**Other (g/kg)**		
Corn starch	404.16	430.49
Maltodextrin 10	134.22	134.22
Sucrose	108.87	108.87
Cellulose, BW200	50.84	50.84
Soybean oil	71.17	71.17
t-Butylhydroquinone	0.01	0.01
Mineral Mix S10022C	3.56	3.56
Calcium carbonate	7.46	7.46
Calcium phosphate, dibasic	7.12	7.12
Potassium phosphate, monobasic	6.98	6.98
Potassium citrate	2.52	2.52
Sodium chloride	2.63	2.63
Vitamin Mix V10037	10.17	10.17
Choline bitartrate	2.54	2.54
Sodium bicarbonate	7.63	7.63
**Kcal (%)**		
Protein	18	15
Carbohydrate	66	69
Fat	16	16
Total Kcal/g	4.0	4.0

## Discussion

To sensitively evaluate the effects on learning and memory based on the behavioral characteristics of experimental animals, appropriate models with minor interindividual variability as well as high learning and memory abilities are required. Since THA rats, which were established through selective breeding for excellent performance in the behavioral avoidance test, showed superior results in various learning-related tests when compared to Wistar rats of the original strain, they are valuable experimental animals in the field of brain research. Therefore, the investigation into the factors that determine the cognitive phenotype of THA rats will allow for elucidating the mechanisms underlying their learning ability and memory.

We employed metabolomic analysis based on the central carbon metabolic pathway in an attempt to obtain a comprehensive understanding of the biological factors involved in learning and memory. The novelty of the present study is the established relationship between learning ability and hippocampal metabolites in THA rats. The high learning ability of THA rats is sustained through constitutively elevated serum levels of BCAA and the activation of BCAA metabolism in the hippocampus. THA rats can rapidly adapt in order to accurately address avoidance learning tasks through the maintenance of greater BCAA metabolism and the subsequent upregulation of ChAT expression and acetylcholine levels in the hippocampus. High levels of serum BCAA in THA rats were explained by the increased expression of amino acid transporter B^0^AT1 in the small intestine and the suppression of BCAA metabolism in the liver. The intake of diets with reduced BCAA content significantly compromised the superior learning ability of THA rats, impairing the cognitive phenotype. Therefore, BCAAs serve as a metabolic basis for the high learning ability and memory of THA rats. Considered together, our findings suggest that maintaining stable BCAA levels can contribute to the prevention and improvement of cognitive decline.

BCAAs Leu, Ile, and Val are three of the nine essential amino acids and are relatively abundant in foods, accounting for 20% of total protein intake^30^. BCAAs are key nitrogen donors in the glutamate/glutamine cycle^31^. Within the CNS, glutamate, which is formed via BCAT-mediated BCAA transamination, is an excitatory neurotransmitter and substrate for the synthesis of the major inhibitory neurotransmitter GABA. Current theories on the role of BCAAs in the brain are consistent with the involvement of glutamatergic and/or GABAergic systems in the etiology of neurological disorders^19,20,22,32^. Fluctuations in BCAA levels significantly influence CNS function, particularly the balance between excitation and inhibition. Since BCAT-mediated metabolism maintains glutamate levels upon the loss of glutamate through oxidation in neurons, BCAA metabolism contributes to brain function homeostasis^32,33^. Of note, the brain’s capacity to oxidize BCAAs is four-fold higher than that of muscles^34^. Therefore, the mammalian brain is essential for the utilization of BCAAs. Several advanced metabolomics studies have indicated that altered BCAA metabolism accompanies Alzheimer’s disease development. Moreover, lower plasma valine levels are correlated with accelerated cognitive decline^35^, while an increase in valine concentration was associated with a reduced risk of Alzheimer’s disease^36^. Cole and colleagues reported that the dietary consumption of BCAAs restored the traumatic brain injury-induced decrease in hippocampal BCAA concentrations in rats, which in turn significantly improved cognitive function^37^. Mice deficient for BCKDK exhibit BCAA hypercatabolism, resulting in abnormally low levels of serum and brain BCAAs. These mutants displayed growth retardation and neurological abnormalities that could be reversed through a BCAA-rich diet^38^. Mutations in the BDKDK gene have been identified in patients with autism spectrum disorders and intellectual disability^39,40^. As the brain depends on a constant supply of BCAAs from the periphery, maintaining BCAA levels in the blood and BCAA metabolism in the brain are critical for the proper CNS function. Together with previous studies, our current findings suggest that increasing the BCAA blood concentrations and metabolism in the brain would be beneficial not only for improving learning and memory but also for the prevention and treatment of neurological disorders. Interestingly, although BCAAs serve as substrates for the synthesis of glutamate and GABA, there is no clear difference between the amounts of either between the hippocampi of Wistar-L, Wistar-H, and THA, indicating that BCAAs are preferably utilized for the synthesis of acetylcholine rather than glutamate and GABA. Acetylcholine plays an important role in memory and has been implicated in dementia, wherein an impairment of hippocampus-dependent learning is observed^41^. In addition, since hippocampal acetylcholine balances the excitatory and inhibitory synaptic inputs onto neurons in step-down inhibitory learning tasks, it plays a pivotal role in the regulation of cognitive functions such as learning and memory^42,43^. Although BCAA-derived acetyl-CoA is generally considered to be utilized for ATP production in the hippocampus, our results strongly suggest that part of the BCAA-derived acetyl-CoA reacts with choline, consequently contributing to acetylcholine synthesis in THA rats. Indeed, the intake of BCAA-reduced diets reduced acetylcholine in the hippocampus and caused a clear decrease in behavioral avoidance test performance. Considered together, our findings indicate that the high learning ability of THA rats is achieved through the rapid activation of BCAA metabolism in the hippocampus, efficiently promoting the production of neurotransmitters involved in learning and memory.

**Figure 5:**
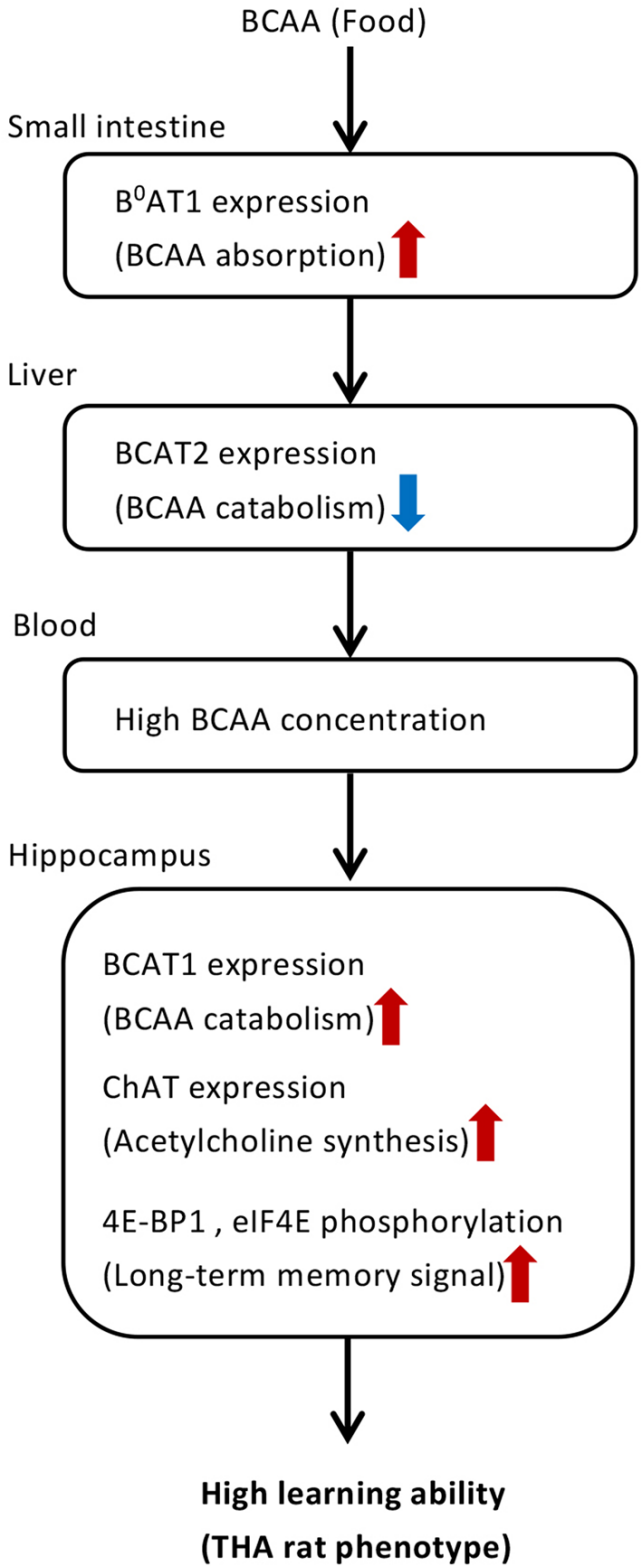
A schematic diagram explaining the high learning ability of THA rats. High levels of BCAAs in the blood of THA rats are due to increased absorption via the small intestine and suppression of BCAA metabolism in the liver. BCAAs are thus metabolized in the hippocampus and involved in the synthesis of acetylcholine, maintaining the high learning ability phenotype in THA rats.

One of the observed mechanisms for maintaining high levels of serum and hippocampal BCAA in THA rats was the increased expression of amino acid transporter B^0^AT1 in the small intestine. Recently, studies have explored the gut-brain axis in an attempt to explain interactions between the CNS and gastrointestinal hormones, which are closely associated with the bidirectional relationship of cognitive disorders with metabolic diseases^44,45^. The vagal efferent nerves, which send signals from the brain to the gut, account for approximately 10–20% of all nerve fibers. The remaining 80% are comprised of vagal afferents carrying information from the gut to the brain^46^. Thus, we assume that THA rats have an optimized mechanism for stimulating B^0^AT1 expression in the gastrointestinal tract and quickly supply BCAAs to the blood through brain-gut or gut-brain axis interactions when BCAA levels are expected to decline. In addition, strong suppression of BCAA catabolism in peripheral tissues, especially the liver, also contributed to the maintenance or increase of serum BCAA levels in THA rats. The first step in BCAA catabolism by BCAT2 occurs primarily within the skeletal muscle after passing through the liver. Further catabolism of α-keto acids is suggested to take place in the liver where the second step, namely oxidation by BCKDHA, takes place^21^. While many researchers believe that there is minor or no expression of BCAT2 in the mammalian liver, sufficient levels of functional BCAT2 have been previously reported in rodent livers^47^, similar to our findings. Further, the liver’s contribution to BCAA oxidation is the third highest after muscle and brown fat, as determined via quantitative analysis of in vivo isotopic tracing^48^. Elective liver transplantation in patients with classical maple syrup urine, which is caused by mutations of BCKDHA and results in the accumulation of BCAAs and their corresponding BCKAs in tissues and plasma, has also been reported to reduce abnormal plasma BCAA levels^49^. Thus, it is evident that the liver plays an important role in BCAA catabolism, further influencing serum BCAA levels that supply the brain. With regard to the concentration of serum BCAA, we found that behavioral avoidance tests markedly suppressed BCKDHA activity in the liver of THA rats and correlated with elevated serum BCAA levels. Although further investigation is needed, THA rats respond to BCAA auxotrophy in the hippocampus through reciprocal regulation of the brain, gut, and liver, thereby sustaining high learning and memory ability (Fig. 5).

Aging is a major causative factor of cognitive decline, and there is great interest in the effective prevention of dementia and other age-related diseases within the field of nutrition. As BCAAs have key physiological roles in the regulation of protein synthesis, metabolism, food intake, and aging, numerous investigations have linked them to age-related outcomes such as sarcopenia, longevity, obesity, and diabetes mellitus^50^. Intriguingly, age-related hippocampus-dependent cognitive decline and muscular weakness were ameliorated in SAMP8 mice, which progressively develop sarcopenia and cognitive decline with an Alzheimer-like phenotype, through the administration of BCAA-enriched diets^51^. Similar to caloric restriction or fasting-mimicking diets, the extension of healthy life span and prevention of multiple diseases associated with energy deficits were also observed in BCAA enriched diets-fed SAMP8 mice. Furthermore, a BCAA-rich diet has been shown to improve clinical outcomes such as cognitive function and muscle performance^52^. The plasma concentrations of BCAAs may influence brain function and affect physical as well as mental fatigue, cognitive performance, physical endurance, sleep, hormonal function, blood pressure, and affective states^53,54^. Opposingly, BCAA restriction has been shown to prevent cognitive decline using the 3xTg-Alzheimer's mouse model^55^, suggesting that the role of BCAAs in cognitive function differs between healthy conditions and model mice predisposed to the disease. Considering previous reports and our current findings, a stable supply of BCAAs and proper metabolic activation within the hippocampus would play important roles in improving cognitive abilities under normal healthy conditions. On the other hand, while the cognitive decline is commonly found towards the end of life, or at least mid-life, our study was conducted only on relatively young and male animals, and further studies are needed to elucidate the role of BCAAs in age-dependent cognitive decline. Future research should consider the effect of BCAAs on the learning function of different ages, multiple animal lines, and both sexes. Furthermore, since each experimental result was obtained from a limited number of animals, increasing statistical output is important to reach more reliable conclusions.

For THA rats, whereas enhancement of BCAA metabolism in the brain plays a crucial role in learning behavior, increased BCAA intake does not always have beneficial effects. Recently, it has been reported that while more BCAAs adversely affect obesity and longevity, BCAA restriction improves mouse frailty and prolongs longevity^56,57^. Yu et al. also suggested that isoleucine has unique adverse effects on metabolism^58^. Blood isoleucine and valine levels have the disadvantage of inversely correlating with lifespan^59^, whereas increased blood valine levels have the advantage of reducing the risk of Alzheimer's disease in humans^36^. Since the roles of BCAAs, such as valine, may be contrastingly different in cognitive and metabolic disorders, the relationship between higher brain function and longevity in individual amino acids needs to be carefully future studied. While BCAAs have diverse roles in human physiology, their significance for higher brain functions such as learning and memory as well as the associated complex regulatory mechanisms have remained unclear. To the best of our knowledge, this is the first study to determine specific molecules that drive the cognitive phenotype of an animal lineage via metabolome analysis. We revealed that retaining high levels of BCAAs in the blood and their utilization for acetylcholine synthesis within the hippocampus is essential for the learning and memory abilities of THA rats. Therefore, this study contributes to elucidating the role of BCAAs in higher brain function, supporting the THA rat as a useful experimental model within neuroscience.

In conclusion, we employed metabolome analysis and revealed that the high learning ability of THA rats was determined using BCAAs. We observed increased BCAA blood levels and enhanced BCAA metabolism within the hippocampus, which were essential for maintaining advanced learning and memory capacities. Our results suggest that BCAA supplementation and/or targeting associated metabolic processes may represent novel approaches for improving cognitive function as a form of therapy against brain dysfunction.

## Methods

### Animals

THA rats of the 110th generation or later were used in this study. THA rats, a Jcl:Wistar rat-derived strain, were selectively bred through brother-sister mating 10-week-old rats under the THA selection protocol^10^. The selection criterion for breeding was an avoidance rate of more than 95% in the lever-pressing test in the latter five sessions of a total of 10 sessions. Jcl:Wistar rats were obtained from CLEA Japan, Inc. (Tokyo, Japan). In order to eliminate the differences in the breeding environment, self-breeding was performed in the same place as the environment in which the THA rats were bred and used in the experiment. The animals were housed at an ambient temperature of 22 ± 1.0 °C, a humidity of 50 ± 10%, and a 12:12-h light–dark cycle environment (lights on between 07:00 and 19:00), with ad libitum access to food (CE-2; CLEA Japan Inc.) and water. All animals used in the present study received humane care, and all experimental procedures were performed in accordance with institutional guidelines and regulations of Tokai University, Japan. All experimental protocols and procedures were reviewed, approved, and carried out in accordance with the guidelines and regulations set by the Animal Experiment Committee of Tokai University (Permit Number 211008, 211009). All experiments were conducted in compliance with the ARRIVE guidelines.

### Experimental design

Figure 1a schematically represents the basic study design and the self-breeding process. Supplementary Fig. S5A online and Fig. 4a show the experiments in which a BCAA-reduced diet was administered starting at 4 or 10 weeks of age. THA rats reared under normal conditions were randomly divided into two groups, a BCAA100 (control group) and a BCAA20 (intervention group). All experimental diets were custom-designed and manufactured in a dry, pelleted form by Research Diets Inc. (Bethlehem, PA, USA). Caloric deficiency due to the decrease in BCAA was corrected through the addition of corn starch. Thus, the diets were isocaloric (4.0 kcal/g) and matched in the total calculated net metabolizable energy (Table 2). The BCAA100 diet had a standard content of BCAAs based on AIN-93G, while the BCAA20 diet contained one-fifth of the standard content of BCAAs.

### Behavioral avoidance test

Male Wistar and THA rats were tested for behavioral avoidance by lever pressing at 5, 10, or 14 weeks of age. Their learning ability was evaluated by a lever-pressing test with the Sidman avoidance schedule^60,61^. Representative situations during the behavioral avoidance test are shown in Supplementary Fig. S1. Eight operant conditioning chambers, each measuring 20.0 × 25.0 × 23.5 cm (length by width by depth), were used. The floor of the chamber was covered with a metal grid with a metal tray beneath. Electric shock generators (Med Associates Inc., St Albans, VT, USA) were connected to the metal grid and used to produce scrambled foot shocks. Each chamber was placed in a sound-attenuated box with a blower fan providing ventilation and background noise (75 dB), and a house-light was placed in the center of the ceiling of the sound-attenuated box^62^. All programming and recording equipment were placed in an adjoining room. This behavioral test was conducted without an exteroceptive warning stimulus^63^. The schedule of the Sidman avoidance test was as follows: electrical aversive shock-to-shock interval, 5 s; lever pressing response-to-shock interval, 30 s; shock intensity DC 100 V 0.7 mA, 0.5 s. Parts of the first and fifth behavioral avoidance learning tests for all sessions are shown (Supplementary Movies 1 and 2, respectively). A maximum of 649 aversive shocks was delivered in each trial. All behavioral avoidance tests were performed for 60 min a day, Monday to Friday. The results of behavioral avoidance tests were expressed as the avoidance rate per hour for each session.

### Sample preparation

Peripheral and portal vein blood was collected the day before the start of the avoidance test and on the day after the end of the test. The rat hippocampus was removed according to a previous method, with minor modifications^64^. Skeletal muscle dissection was then performed to harvest the gastrocnemius muscle from the same location^65^. BAT was obtained from between the scapulas according to a previously reported protocol^66^ and carefully removed due to the attachment of other subcutaneous tissue to the BAT. Livers were collected from the right middle lobe to ensure reproducibility of the results^67^. Since the ileum has the highest expression of B^0^AT1 among small intestinal segments, the small intestine analyzed was defined as the ileum 10 cm from the cecum to the stomach as per a previously reported protocol^68^. The tissues were collected prior to the avoidance test or after 10 sessions of testing and were quickly dissected, immediately placed in liquid nitrogen, and deep-frozen (− 80 °C) until metabolome analysis or biochemical tests.

### Metabolome analysis

Briefly, approximately 30 mg of frozen hippocampus was homogenized in 50% acetonitrile/Milli-Q water and centrifuged. The upper aqueous layer was filtered through a Millipore 5 kDa cutoff filter to remove proteins prior to CE-MS analysis. A total of 116 targeted metabolites engaged in central metabolism were measured with absolute quantification using the C-SCOPE package (Human Metabolome Technologies (HMT), Inc., Tsuruoka, Japan) as described previously^69,70^. Capillary electrophoresis time-of-flight mass spectrometry (CE-TOFMS) was used for cation analysis while CE-tandem mass spectrometry (CE-MS/MS) for anion analysis^71–74^. Briefly, CE-TOFMS and CE-MS/MS were carried out using an Agilent CE capillary electrophoresis system equipped with an Agilent 6210 time-of-flight mass spectrometer (Agilent Technologies, Waldbronn, Germany) and Agilent 6460 Triple Quadrupole LC/MS, respectively. The systems were controlled by Agilent G2201AA ChemStation software version B.03.01 for CE (Agilent Technologies) and connected by a fused silica capillary (50/µm i.d. × 80 cm total length) with a commercial electrophoresis buffer (H3301-1001 and H3302-1021 for cation and anion analyses, respectively, HMT) as the electrolyte. The time-of-flight mass spectrometer was scanned from m/z 50 to 1000, and the triple quadrupole mass spectrometer was used to detect compounds in dynamic MRM mode. Peaks were extracted using MasterHands, automatic integration software version 2.16.0.15 (Keio University, Tsuruoka, Japan)^74^, and MassHunter Quantitative Analysis B.06.00 (Agilent Technologies, Santa Clara CA) in order to obtain peak information, including m/z, peak area, and migration time (MT). Signal peaks were annotated according to the HMT metabolite database based on their m/z values with MTs. In the HMT system, quality was controlled using the coefficient of variation (CV) calculated from the peak area of the internal standard in each sample. The CV value threshold was set to 15% or less, and all samples were confirmed to be below the threshold. The peak area of each metabolite was normalized with respect to the area of the internal standard, and metabolite concentrations were determined using standard curves with three-point calibrations using each standard compound. HCA and PCA were performed using HMT proprietary software PeakStat version 3.18 and SampleStat version 3.14 (HMT, https://humanmetabolome.com), respectively.

### Biochemical measurements of metabolites

The concentration of BCAA in the serum and hippocampus was determined using a Branched Chain Amino Acid Assay Kit (Sigma-Aldrich, St. Louis, MO, USA), according to the manufacturer's instructions. The level of acetylcholine in the brain was measured using the Amplite Fluorimetric Acetylcholine Assay Kit (AAT Bioquest Inc., Sunnyvale, CA, USA) according to the manufacturer’s protocol. Since this kit detects both acetylcholine and choline, the former’s levels were obtained by subtracting the values obtained with the Amplite Choline Quantitation kit (AAT Bioquest Inc.), which detects only choline. All samples were deproteinized via centrifugal Millipore ultrafiltration (10-kDa cutoff, Millipore Corp, Bedford, USA) prior to metabolite analyses. Absorbance and fluorescence intensity was measured using a SpectraMax I3 microplate reader (Molecular Devices, Sunnyvale, CA, USA).

### Western blot analysis

Preparation of total protein extracts from tissues, electrophoresis, and subsequent blotting was performed as previously described^75–77^. Membranes were probed with antibodies against BCKDH-E1α, phospho-BCKDH-E1α (Ser293), ChAT, GAPDH, 4E-BP1, phospho-4E-BP1 (Thr37/46), eIF4E, phospho-eIF4E (Ser209), S6 ribosomal protein, phospho-S6 ribosomal protein (Ser235/236) (Cell Signaling Technology, Beverly, MA, USA), BCKDK (Sigma-Aldrich), BCAT1/ECA39, BCAT2 (Proteintech Group, Chicago, IL, USA), SLC6A19 (B^0^AT1) (Abcam, Cambridge, UK), and β-actin (MBL, Nagoya, Japan).

### Statistical analysis

Data are expressed as the mean ± SD. For the metabolome analysis via CE-TOFMS, data analysis was performed using Welch’s *t*-test, according to the manufacturer’s suggestions. Two groups were compared using the Student’s *t*-test (two-tailed) for parametric data or the Mann–Whitney *U* test for non-parametric data in GraphPad Prism 8.0 (GraphPad Software, La Jolla, CA, USA). Statistical significance was set at *P* < 0.05.

## Supplementary Information

Supplementary Figures.

Supplementary Video 1.

Supplementary Video 2.

## Data Availability

The datasets generated during and/or analyzed during this study are available from the corresponding author upon reasonable request.
