# Supplementary material for: Branched-chain amino acids govern the high learning ability phenotype in Tokai high avoider (THA) rats
Source: Sci Rep. 2021 Nov 29;11:23104. doi: 10.1038/s41598-021-02591-7 (PMC8630195; doi:10.1038/s41598-021-02591-7)

Supplementary Information

Branched-Chain Amino Acids Govern the High Learning Ability Phenotype in Tokai High Avoider (THA) Rats

Yukari Shida [a, #], Hitoshi Endo [a, # *], Satoshi Owada [a], Yutaka Inagaki [b], Hideaki Sumiyoshi [b], Akihide Kamiya [c], Tomoo Eto [d], and Masayuki Tatemichi [a].

[a] Center for Molecular Prevention and Environmental Medicine, Department of Preventive Medicine, Tokai University School of Medicine, 143 Shimokasuya, Isehara, Kanagawa 259-1193, Japan

[b] Center for Matrix Biology and Medicine, Department of Innovative Medical Science, Tokai University School of Medicine, 143 Shimokasuya, Isehara, Kanagawa 259-1193, Japan

[c] Department of Molecular Life Sciences, Tokai University School of Medicine, 143 Shimokasuya, Isehara, Kanagawa 259-1193, Japan

[d] Central Institute for Experimental Animals, 3-25-12 Tonomachi, Kawasaki-ku, Kawasaki Kanagawa 210-0821, Japan

[#]These authors contributed equally to this work.

[*]Correspondence:

Hitoshi Endo, Ph.D.

Center for Molecular Prevention and Environmental Medicine, Department of Preventive Medicine, Tokai University School of Medicine, 143 Shimokasuya Isehara, Kanagawa 259-1193, Japan

Phone: +81-463-93-1121, Fax: +81-463-92-3549

E-mail: h-endo@tokai-u.jp

Supplementary figures and legends:

Figure S1. Image of the behavioral avoidance test wherein electrical stimulation is avoided by pressing the lever.

Figure S2. Comparison of essential amino acid levels in the hippocampus of Wistar-L (n = 3), Wistar-H (n = 3), and THA (n = 4) rats via metabolome analysis. Data are presented as mean ± SD ***P < 0.001.

Figure S3. (A) Comparison of glycolytic metabolite levels in the hippocampus as well as the levels of (B) ATP, (C) glutamate, and (D) GABA between Wistar-L (n = 3), Wistar-H (n = 3), and THA (n = 4) rats via metabolome analysis. Data are presented as the mean ± SD *P < 0.05.

Figure S4. Comparison of small intestinal B^0AT1 expression and portal or peripheral blood BCAA levels between Wistar and THA rats. (A) BCAT2, P-BCKDHA, T-BCKDHA expression in the small intestine of Wistar and THA rats after the avoidance test (Wistar rats n = 3, THA rats n = 3). (B) B^0AT1 expression in the small intestine (Wistar rats n = 3, THA rats n = 3) and serum from (C) portal vein blood or (D) peripheral blood in Wistar and THA rats before the avoidance test (Wistar rats n = 4, THA rats n = 4). (E) Comparison of liver of Wistar or THA rats before (BF test) and after (AF test) the avoidance test (Wistar rats n = 4, THA rats n = 4). Western blot analyses of BCAT2, P-BCKDHA, and T-BCKDHA and their quantified ratios, with β-actin as a loading control. In Figure S4A, β-actin in Figure 3E was the loading control for the same sample. Data are expressed as the mean ± SD values. * P < 0.05; ** P < 0.01.

Figure S5. Effects of BCAA-restricted diets at a young age and differences in learning ability between the young and mature stages of THA rats. (A) Schematic representation of the

experimental flow with a BCAA-reduced diet. BCAA100: control, BCAA20: one-fifth BCAA of control. (B) Serum BCAA concentration in BCAA100 (n = 3) and BCAA20 (n = 6) groups after avoidance tests. Changes in (C) dietary intake and (D) body weight per animal after 4 weeks of age in the BCAA100 and BCAA20 groups, respectively. (E) Comparison of avoidance rate during the lever-pressing behavioral avoidance tests at 5 and 10 weeks of age (5 weeks n = 4, 10 weeks n = 3). (F) Comparison of hippocampi in THA rats before (BF test, n = 3) and after (AF test, n = 3) the avoidance test. Western blot analyses P-BCKDHA and T-BCKDHA and their quantified ratios. β-actin served as a loading control. Changes in (G) dietary intake and (H) body weight per animal after 10 weeks of age in the BCAA100 (n = 6) and BCAA20 (n = 8) group, respectively. Data are expressed as the mean ± SD values. ** $P < 0.01$.

Figure S6. Uncropped Western blots for all the immunoblotting data presented in the main figures and supplementary figures are shown. The full-length membrane was cut and incubated with the indicated antibody. Boxed regions are used in the figures.

Supplementary Movie 1. Example of a lever-pressing behavioral avoidance test based on a Sidman schedule. A part of the first session for a THA rat is shown.

Supplementary Movie 2. A part of the fifth session for a THA rat is shown. This THA rat is the same individual as the one in Supplementary Movie 1.

Supplementary figure 1

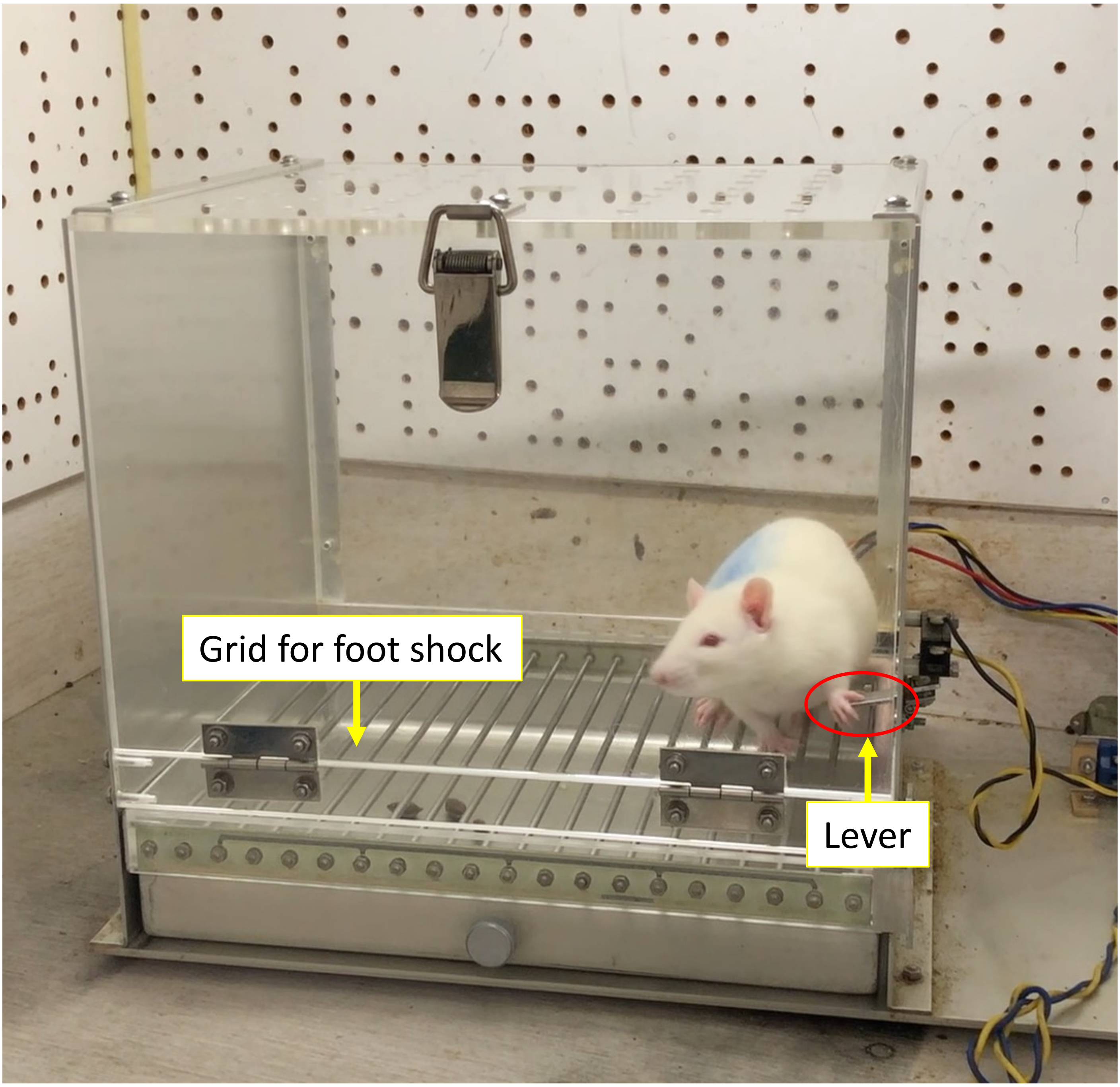

Supplementary figure 2

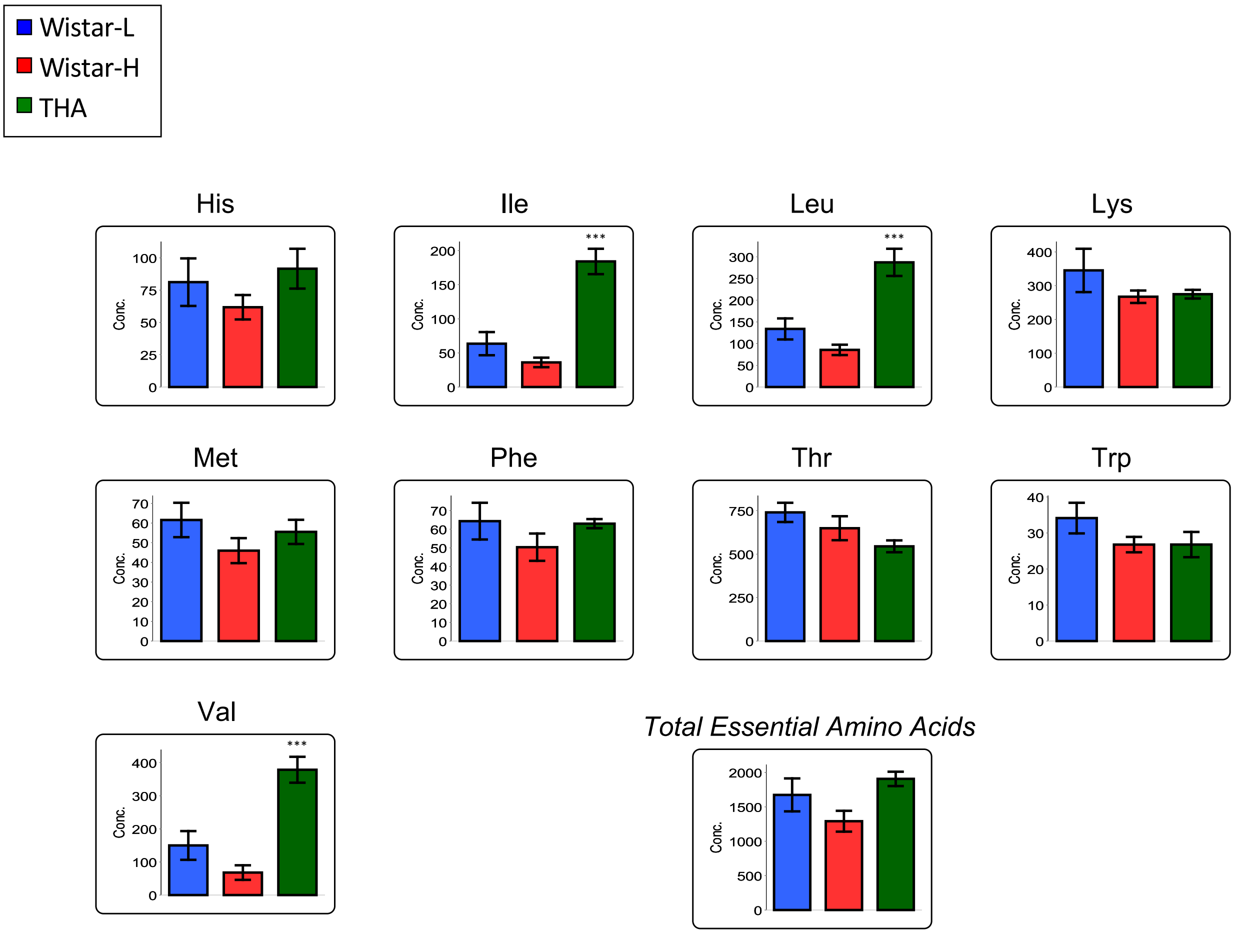

Supplementary figure 3

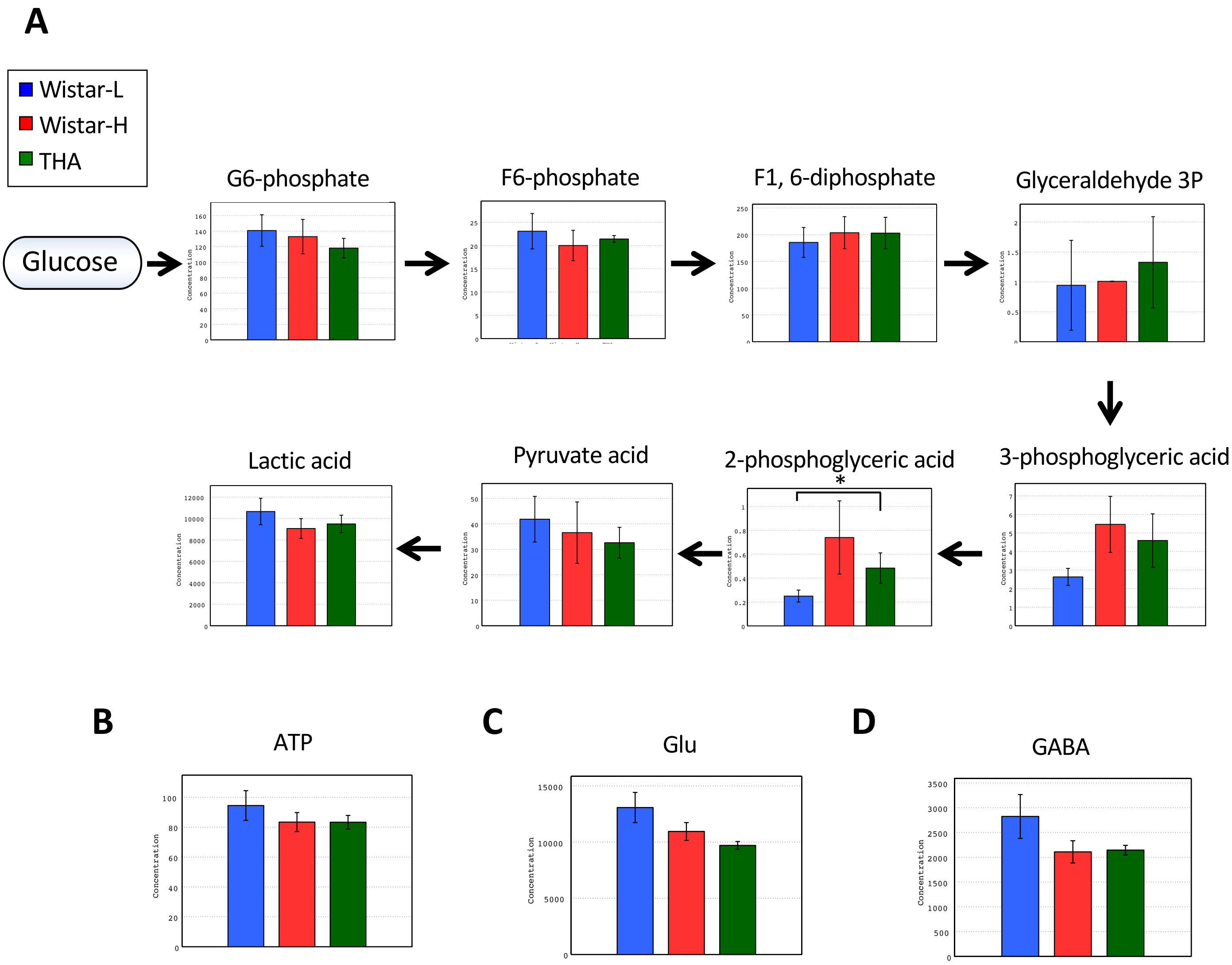

Supplementary figure 4

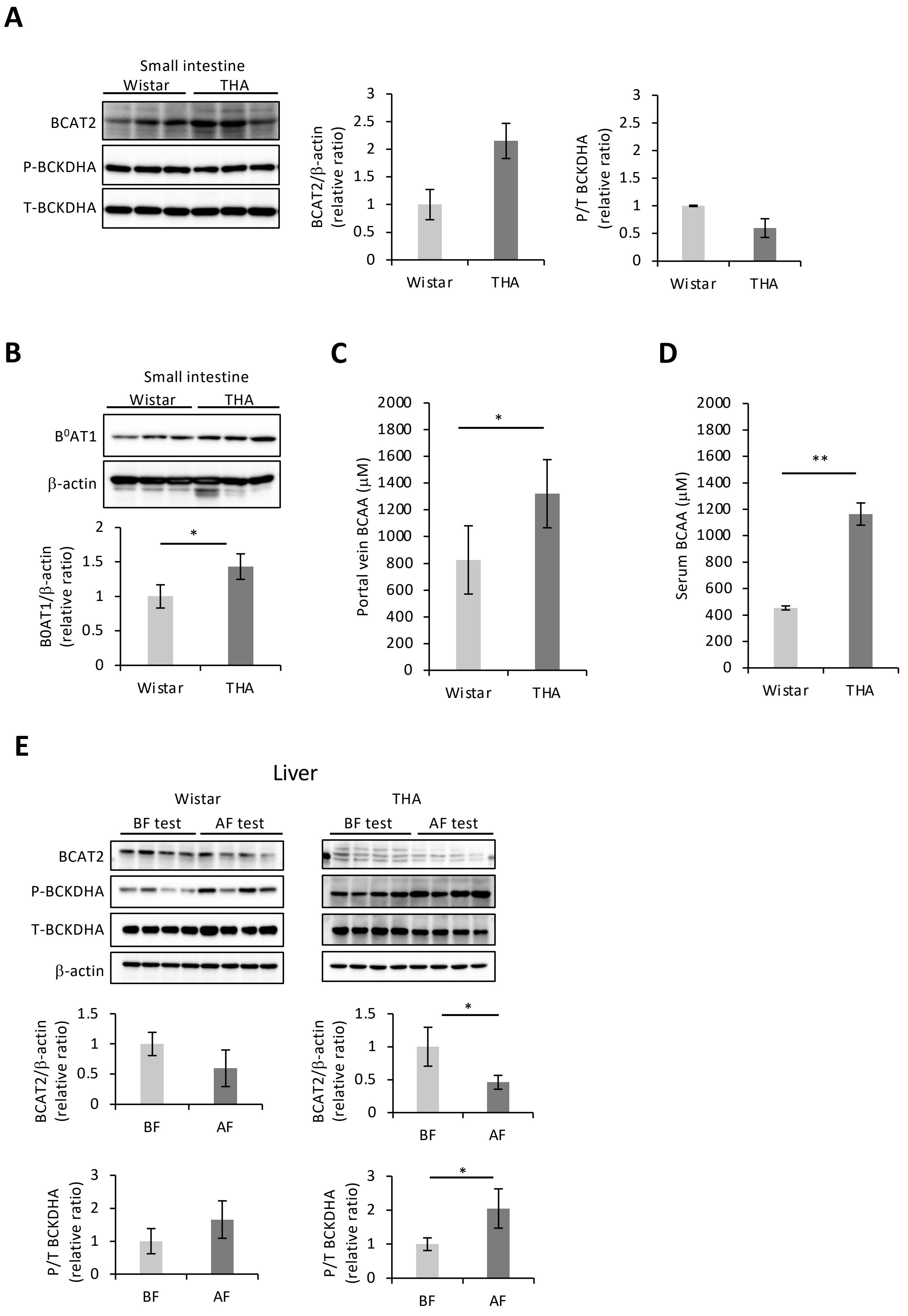

Supplementary figure 5

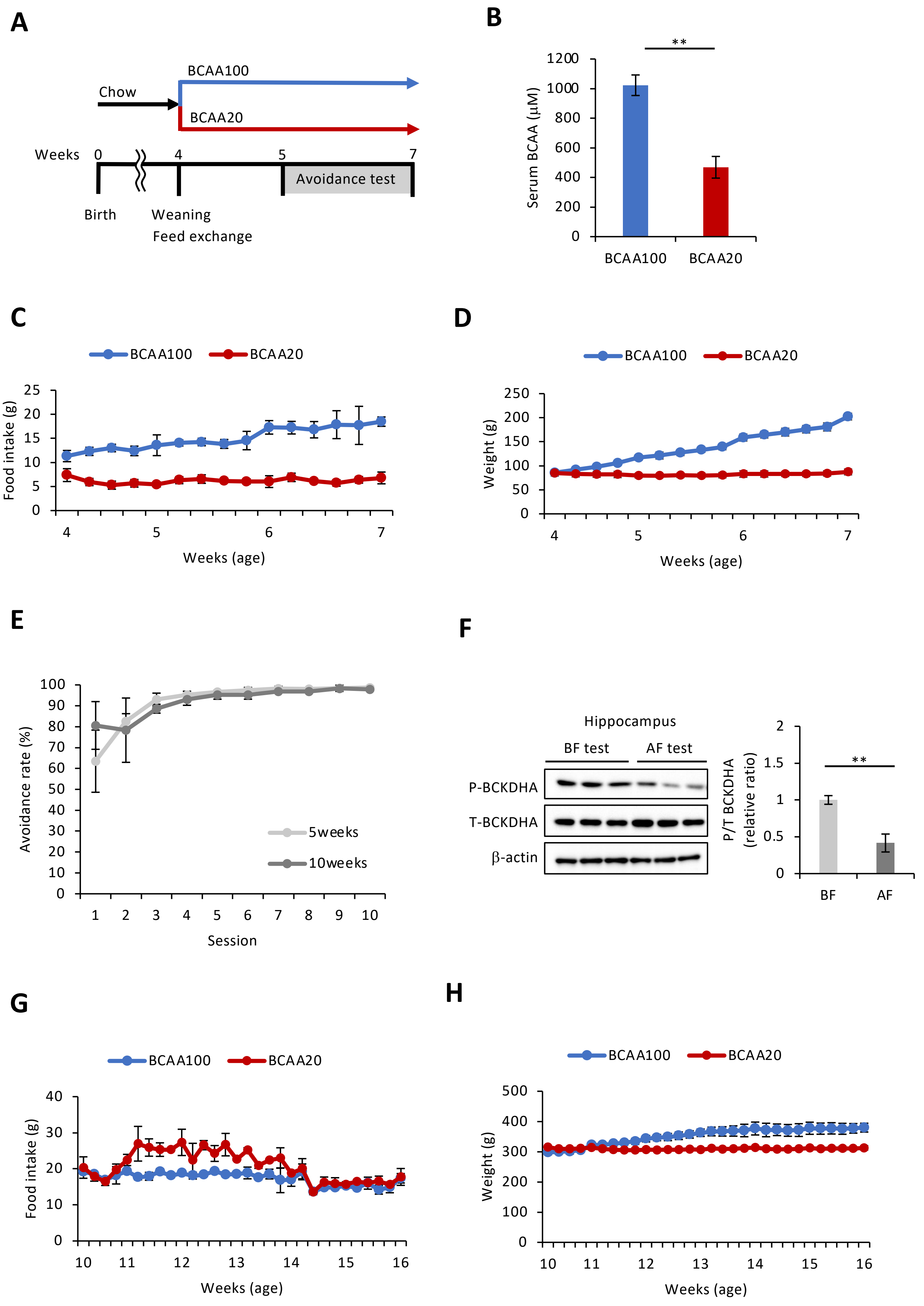

Supplementary figure 6

Figure 2: Full membrane western blot data

b

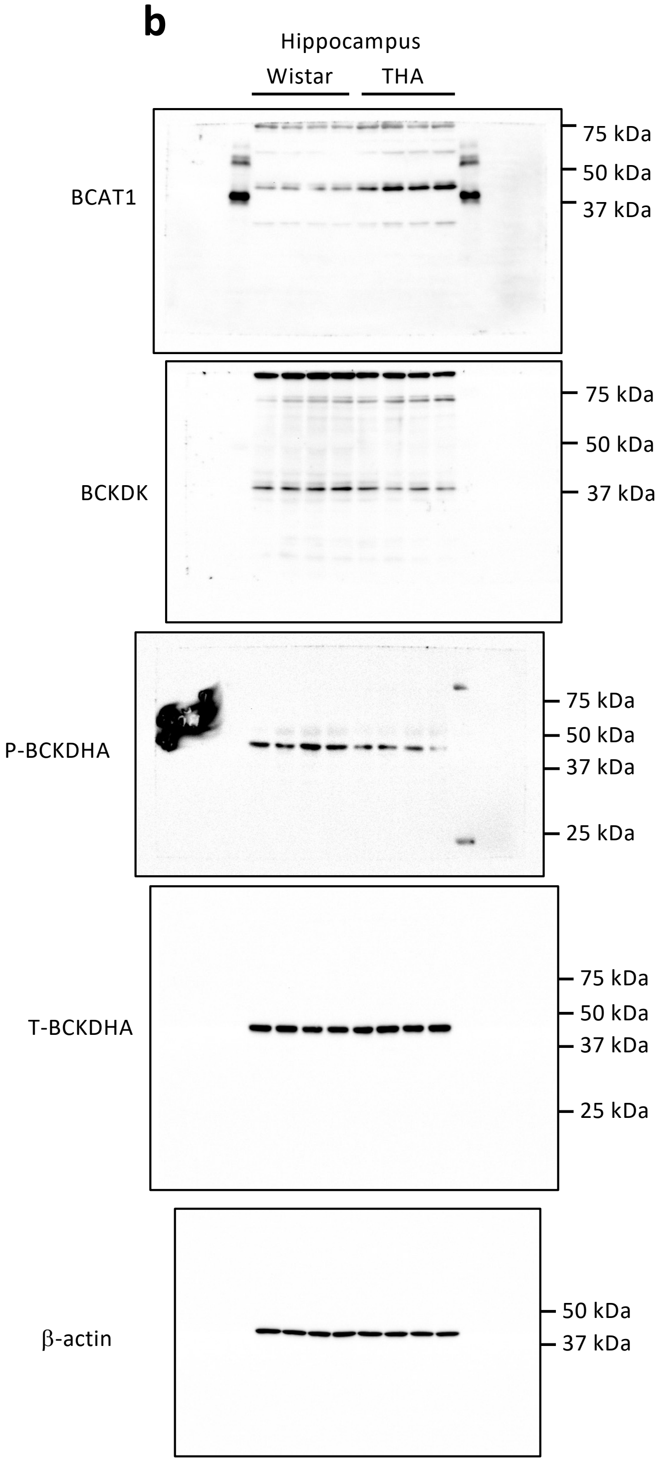

e

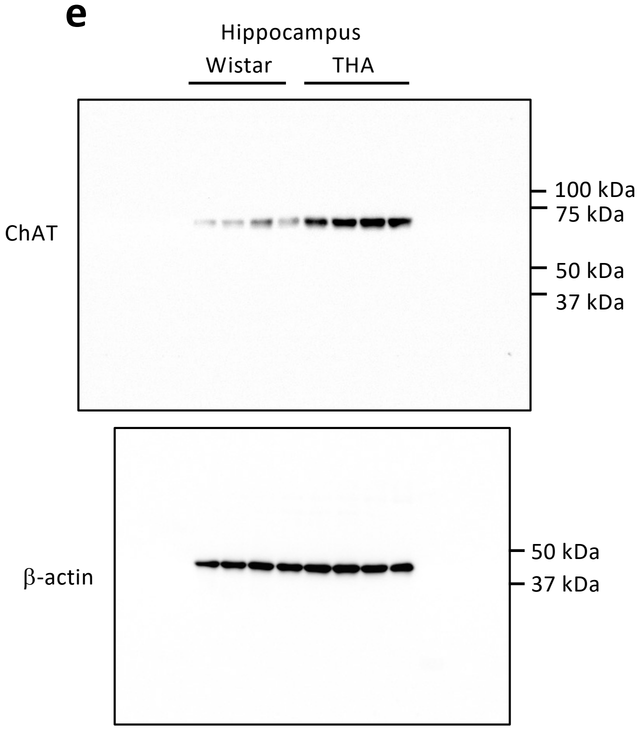

Continued supplementary figure 6

Figure 2: Full membrane western blot data

g

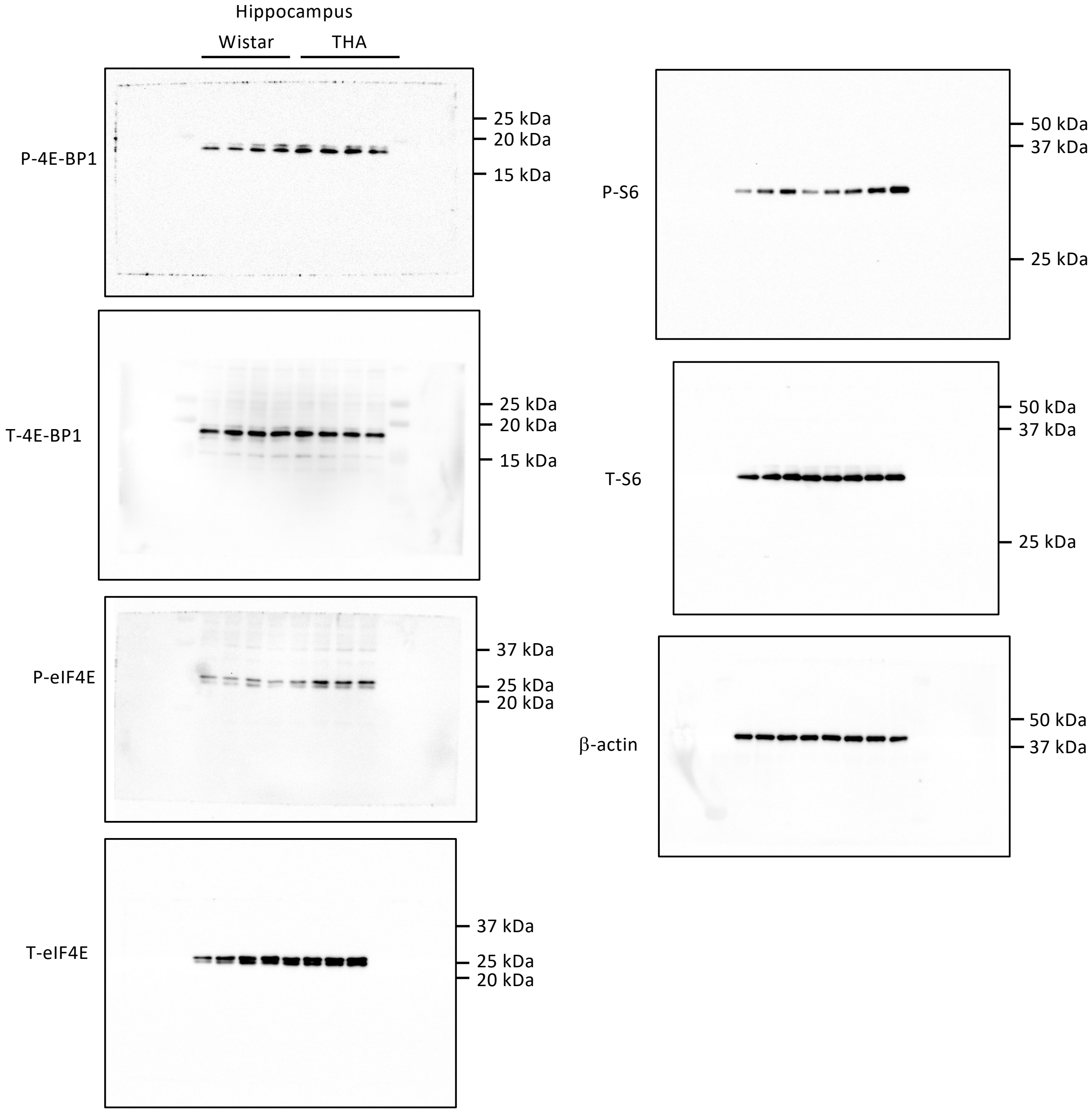

Continued supplementary figure 6

Figure 2: Full membrane western blot data

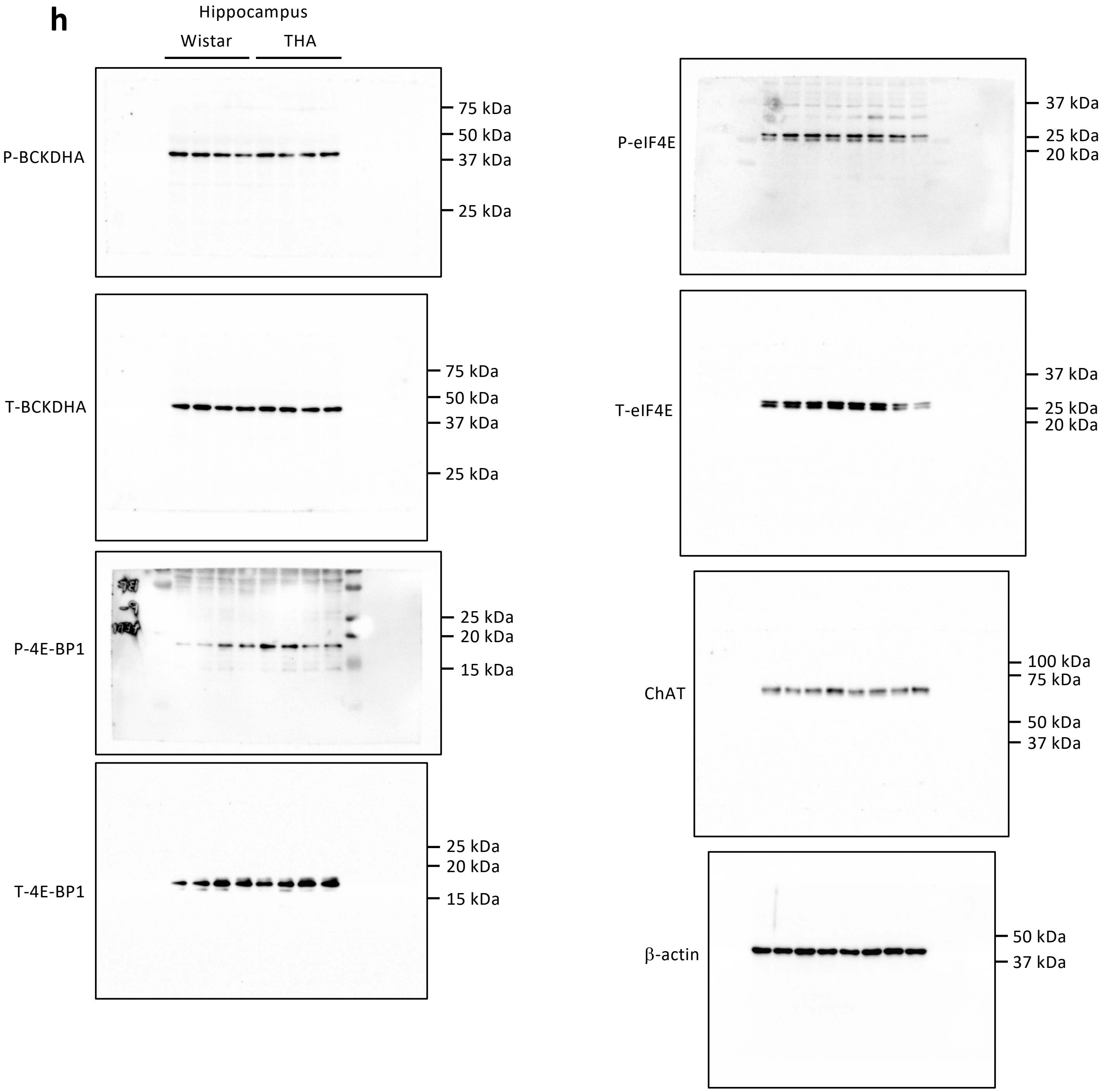

Continued supplementary figure 6

Figure 2: Full membrane western blot data

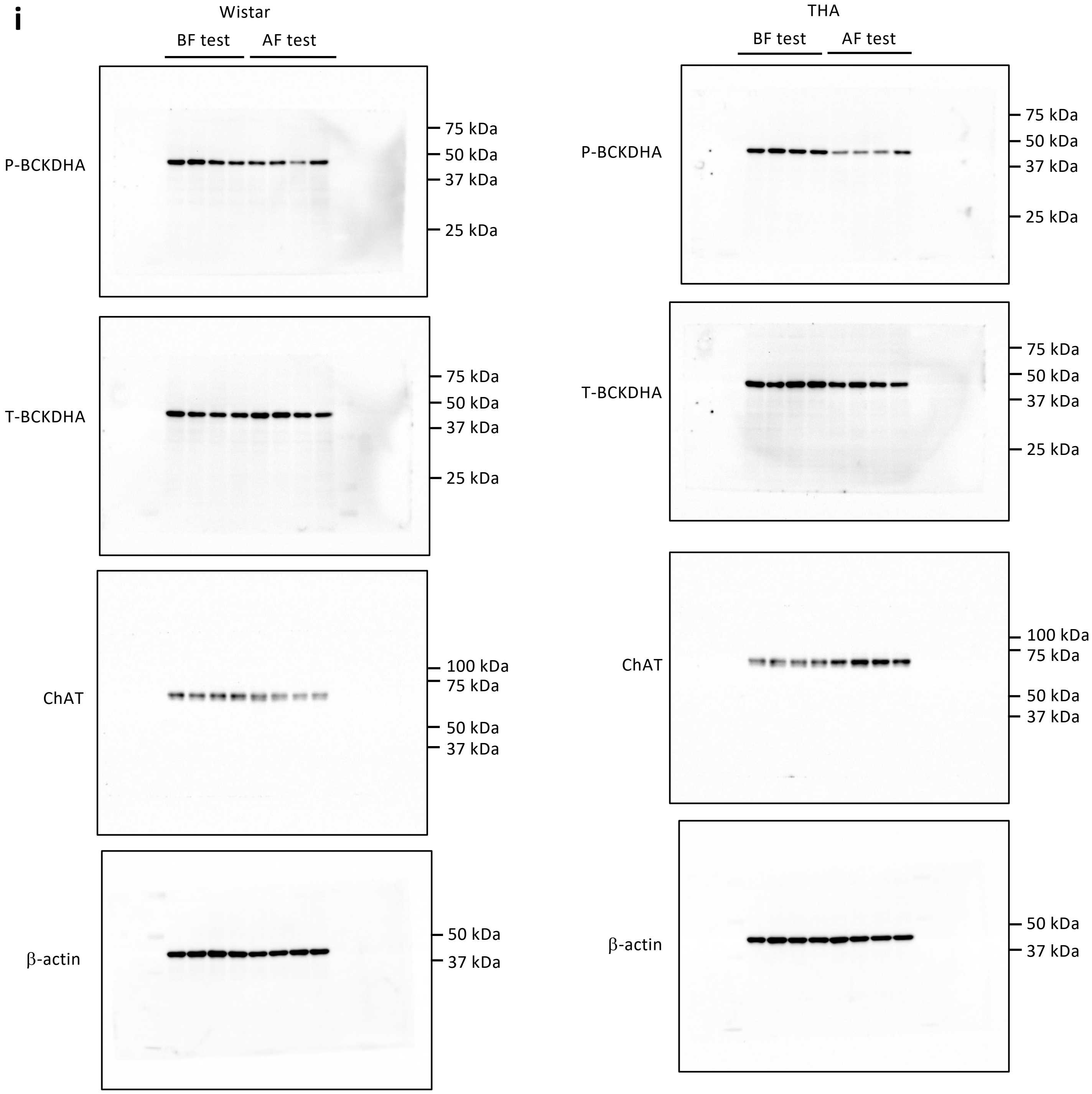

Continued supplementary figure 6

Figure 3: Full membrane western blot data

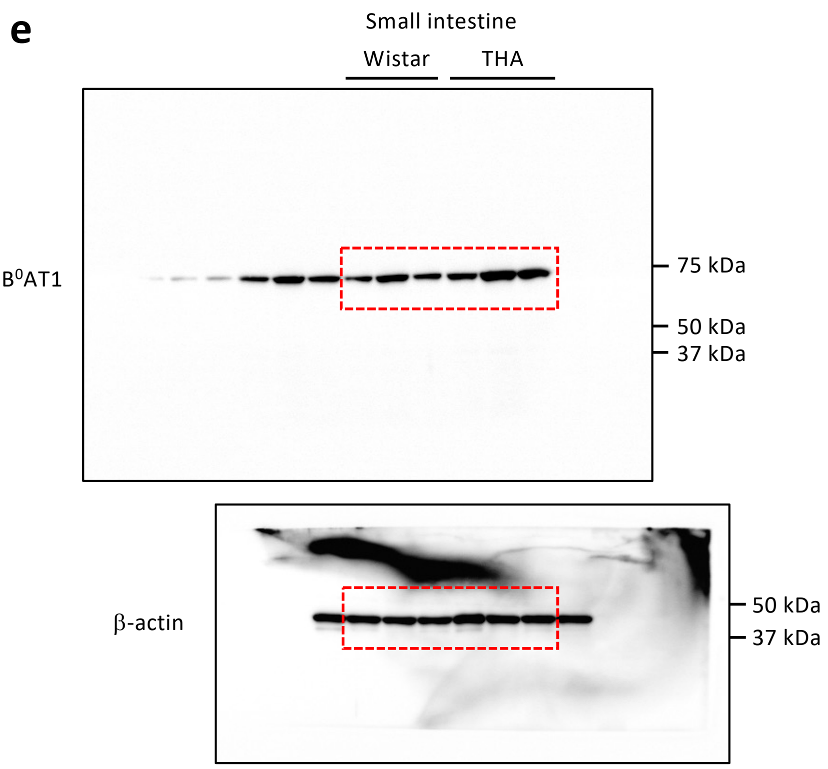

Continued supplementary figure 6

Figure 3: Full membrane western blot data

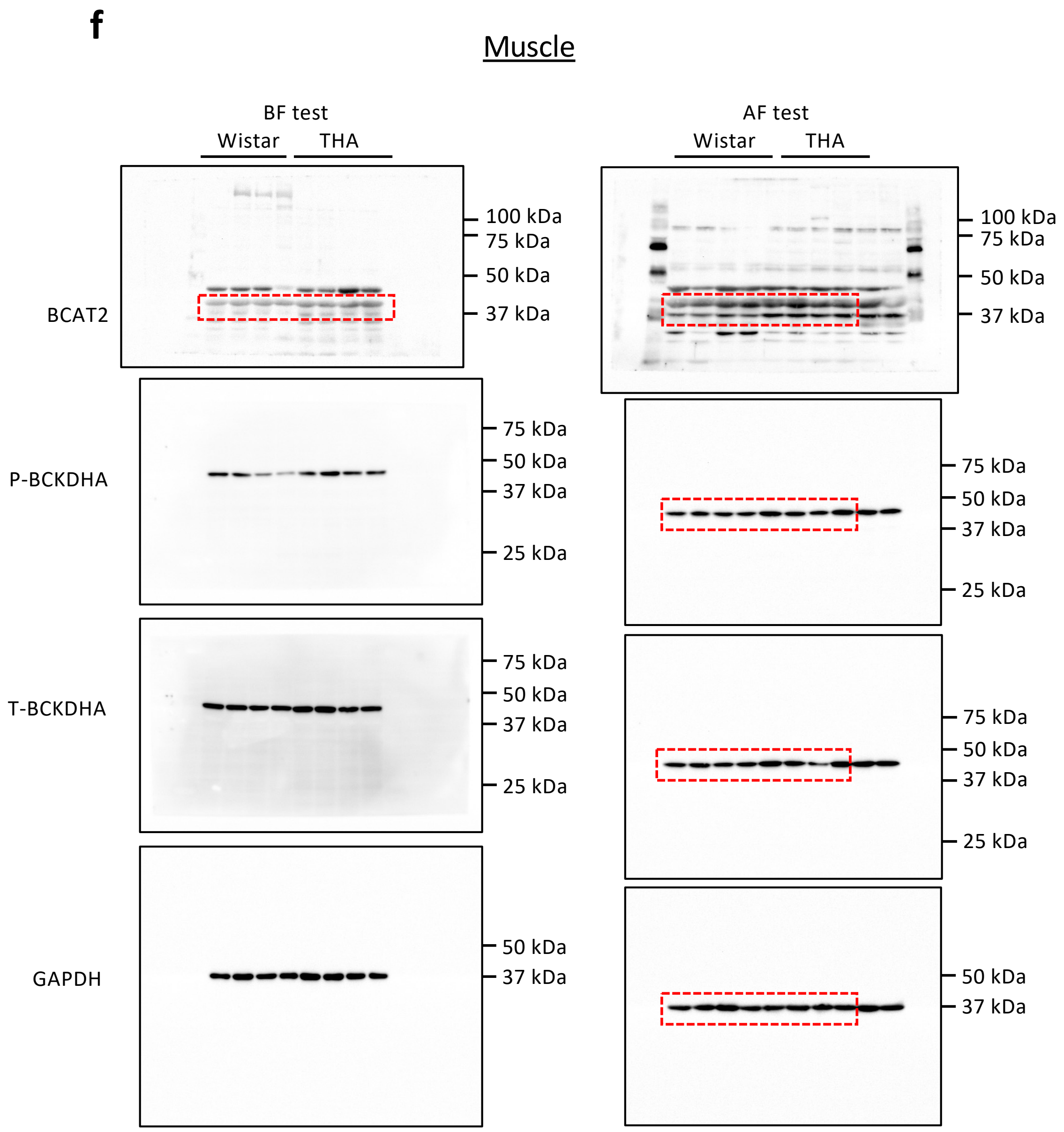

Continued supplementary figure 6

Figure 3: Full membrane western blot data

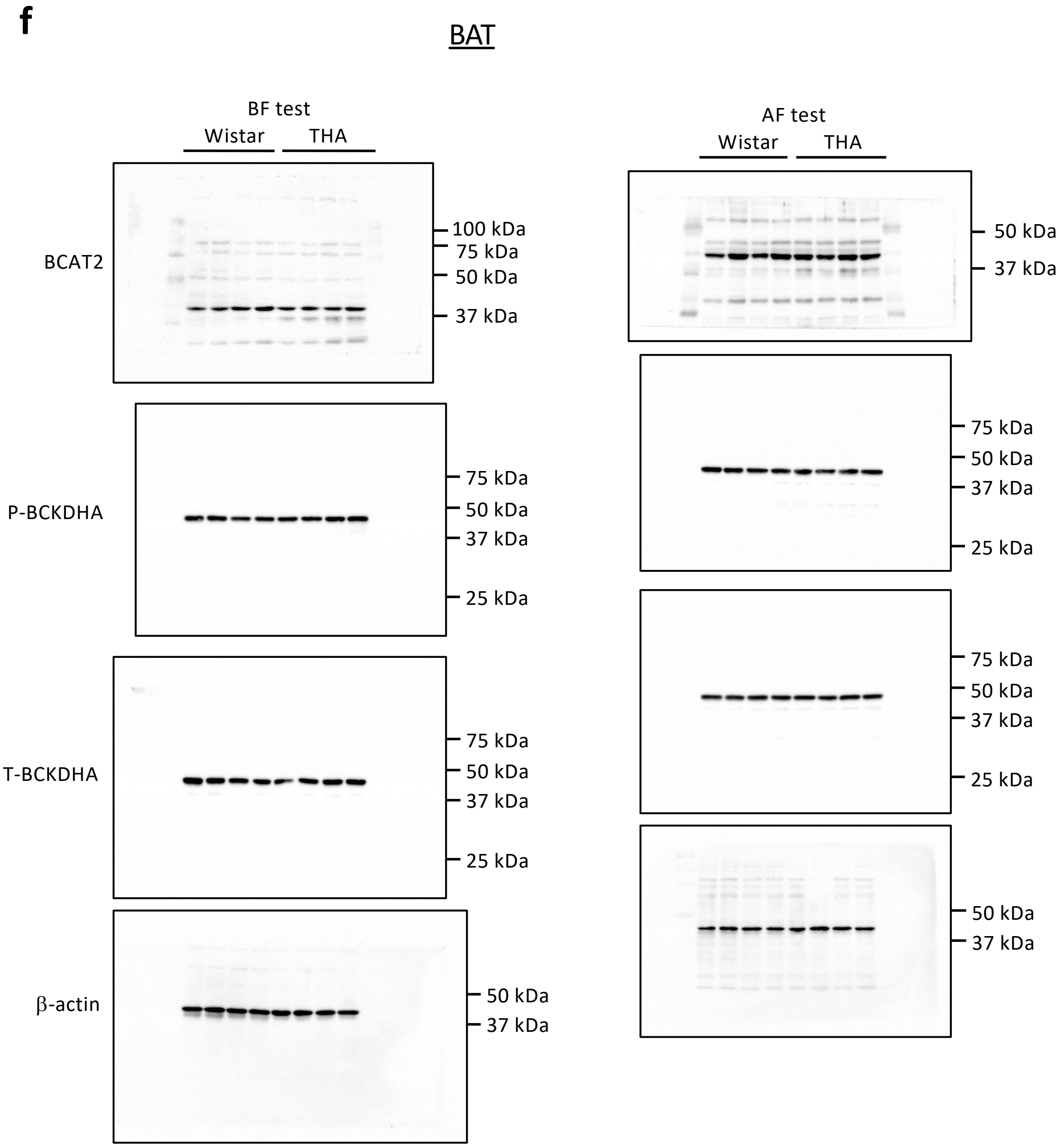

Continued supplementary figure 6

Figure 3: Full membrane western blot data

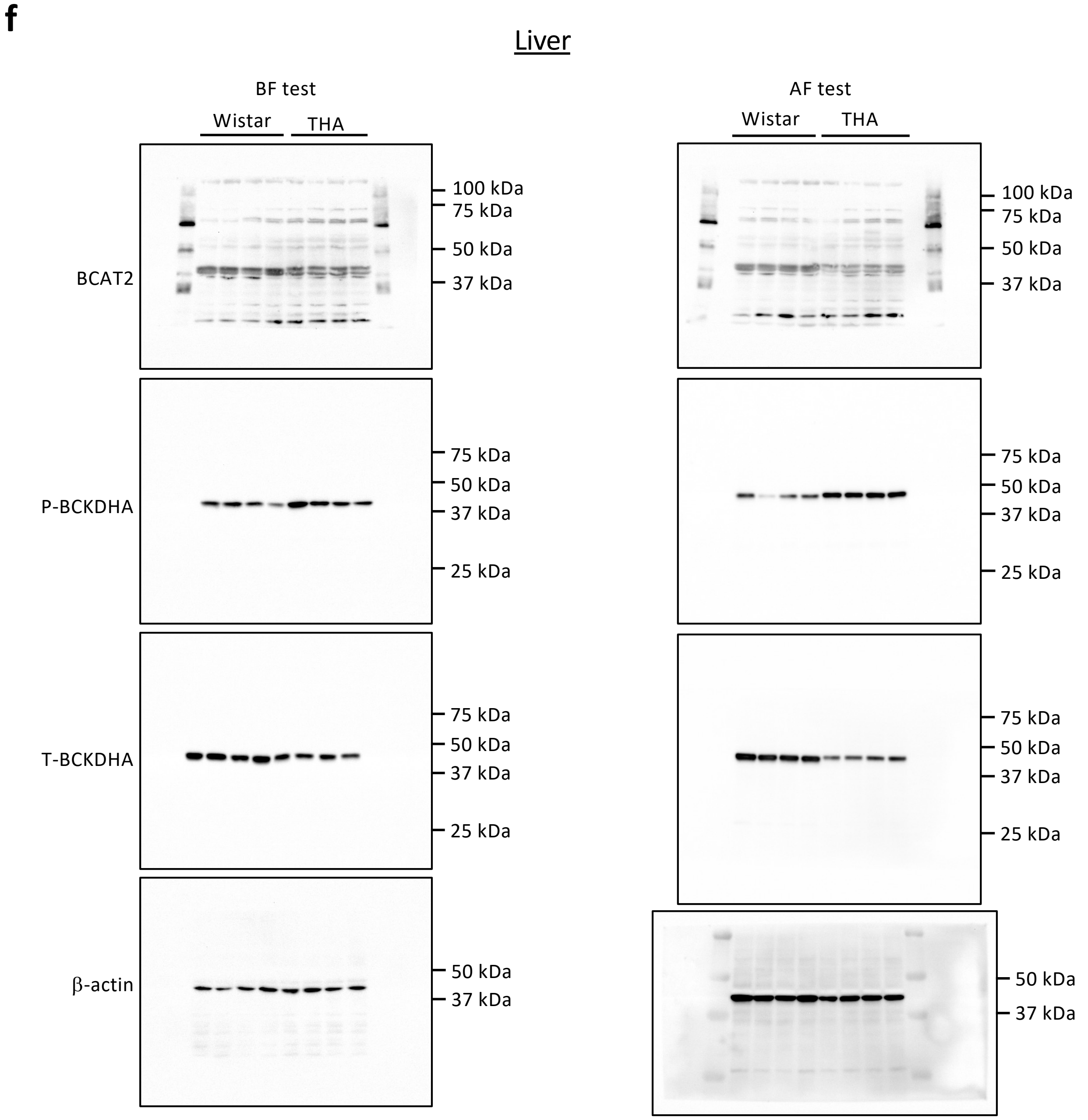

Continued supplementary figure 6

Figure4: Full membrane western blot data

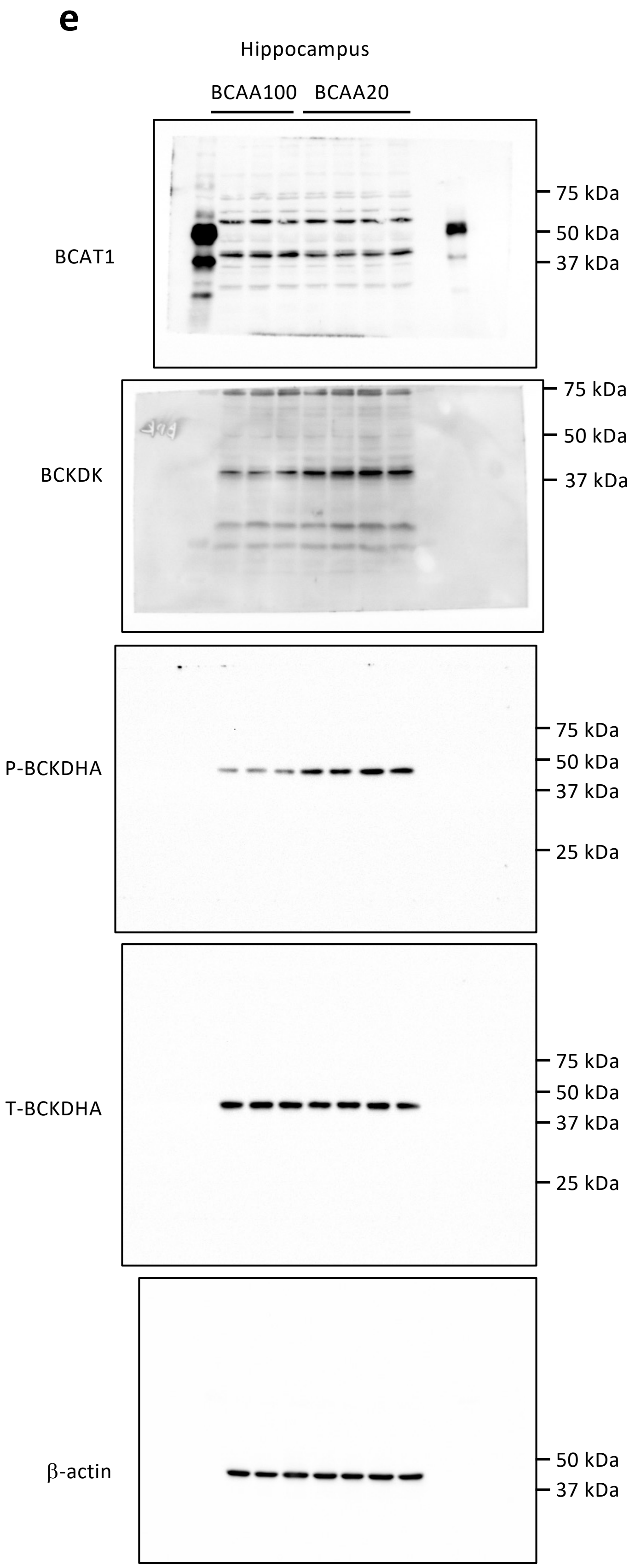

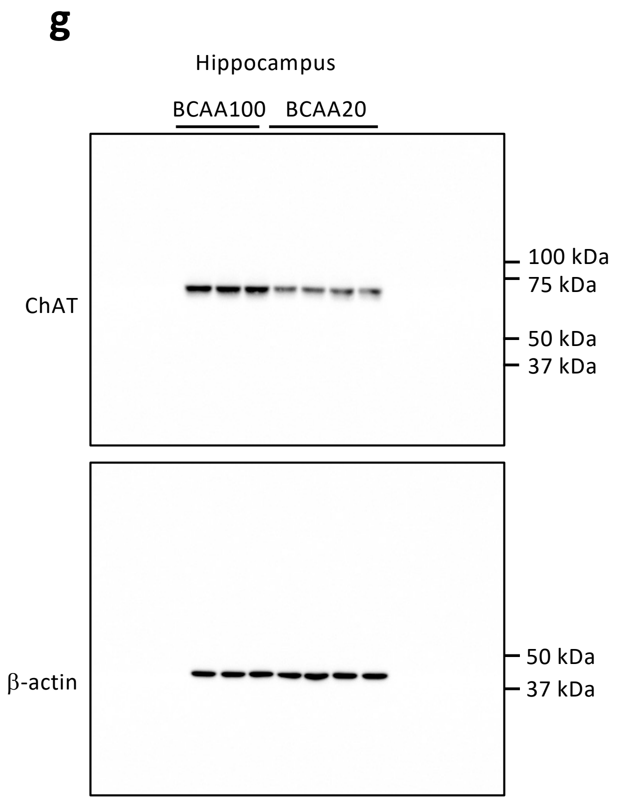

Continued supplementary figure 6

Figure4: Full membrane western blot data

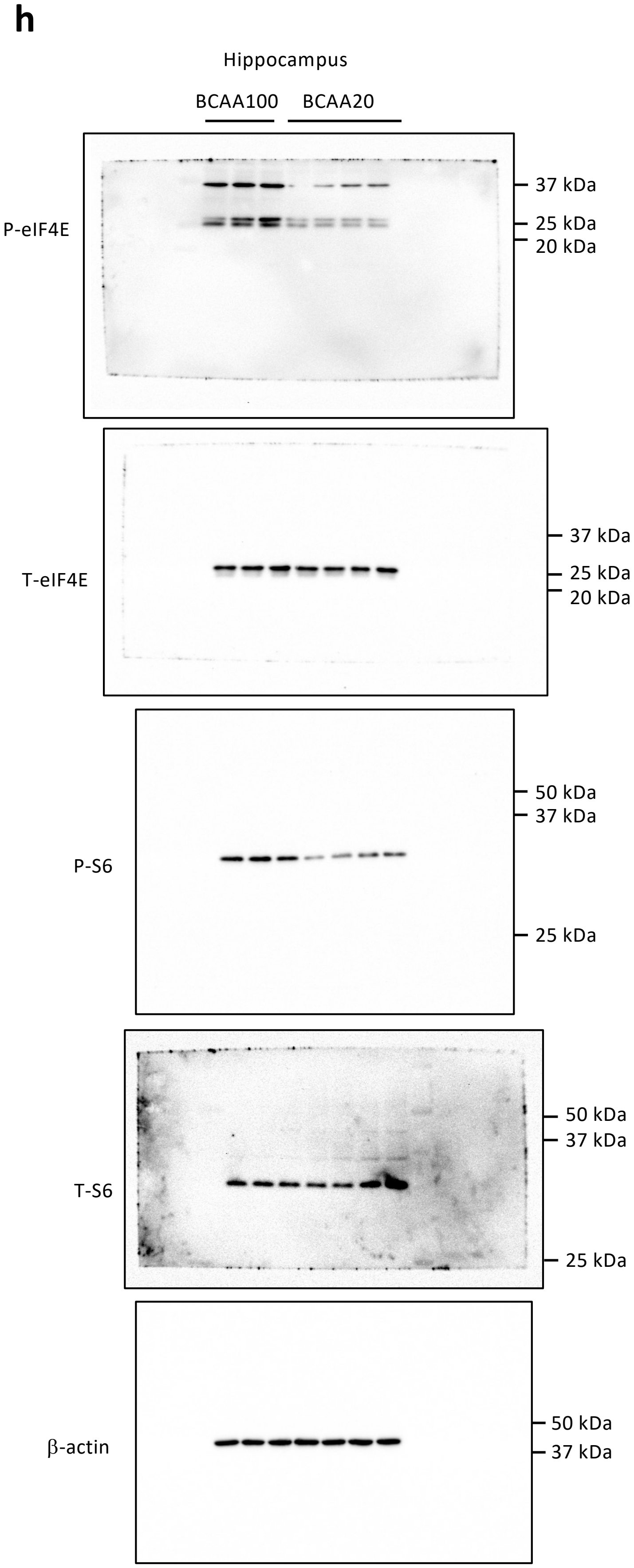

Continued supplementary figure 6

Supplementary figure 4: Full membrane western blot data

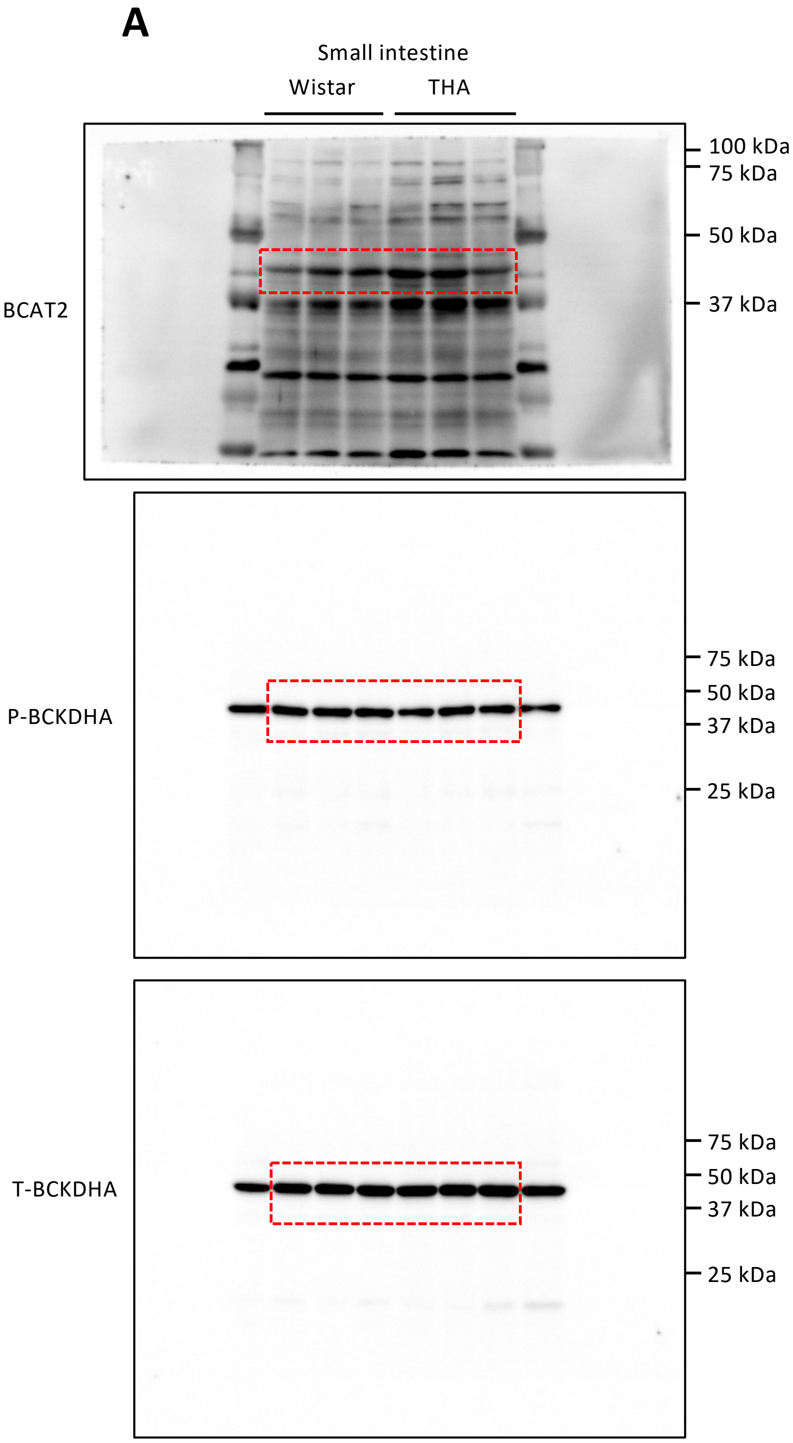

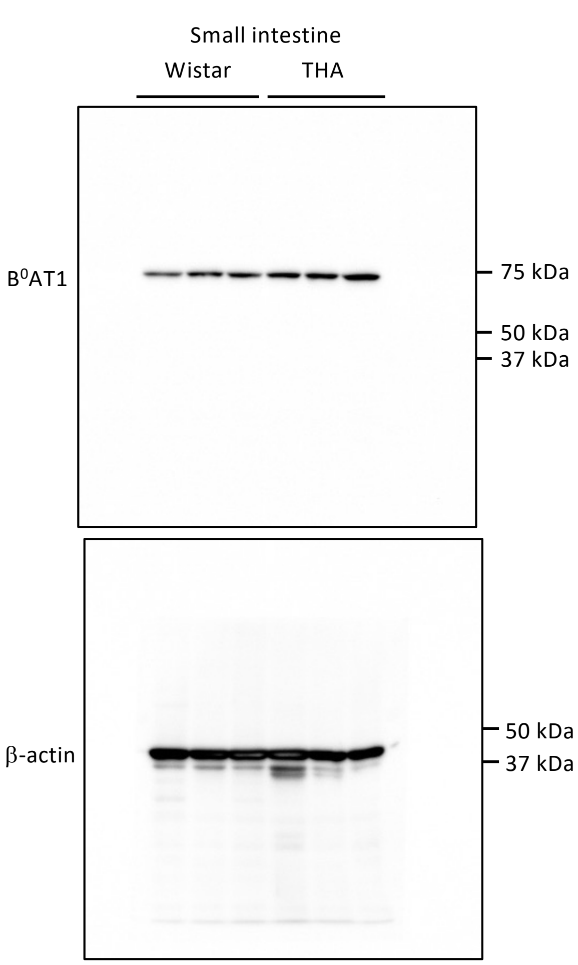

Continued supplementary figure 6

Supplementary figure 4: Full membrane western blot data

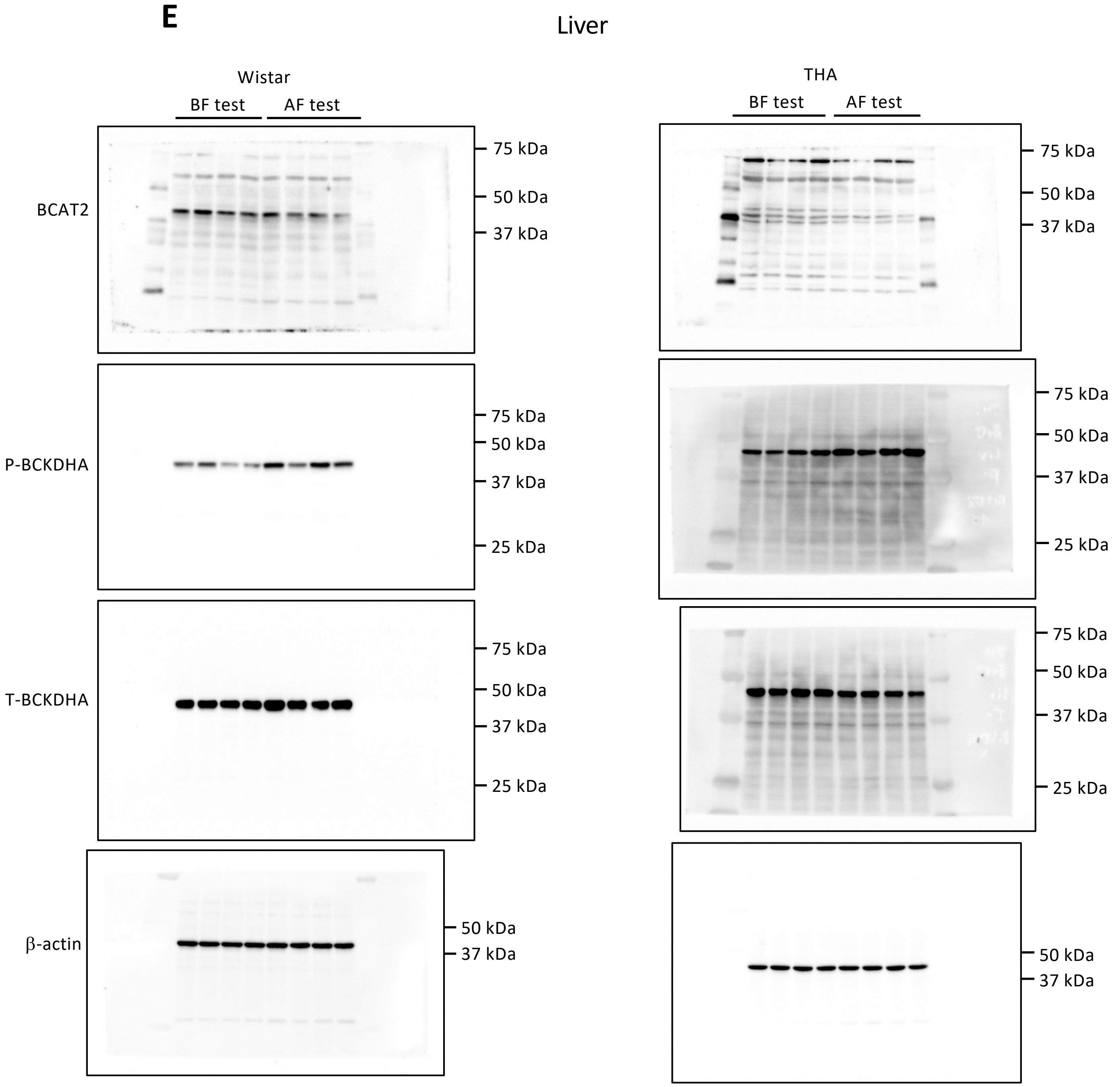

Continued supplementary figure 6

Supplementary figure 5: Full membrane western blot data

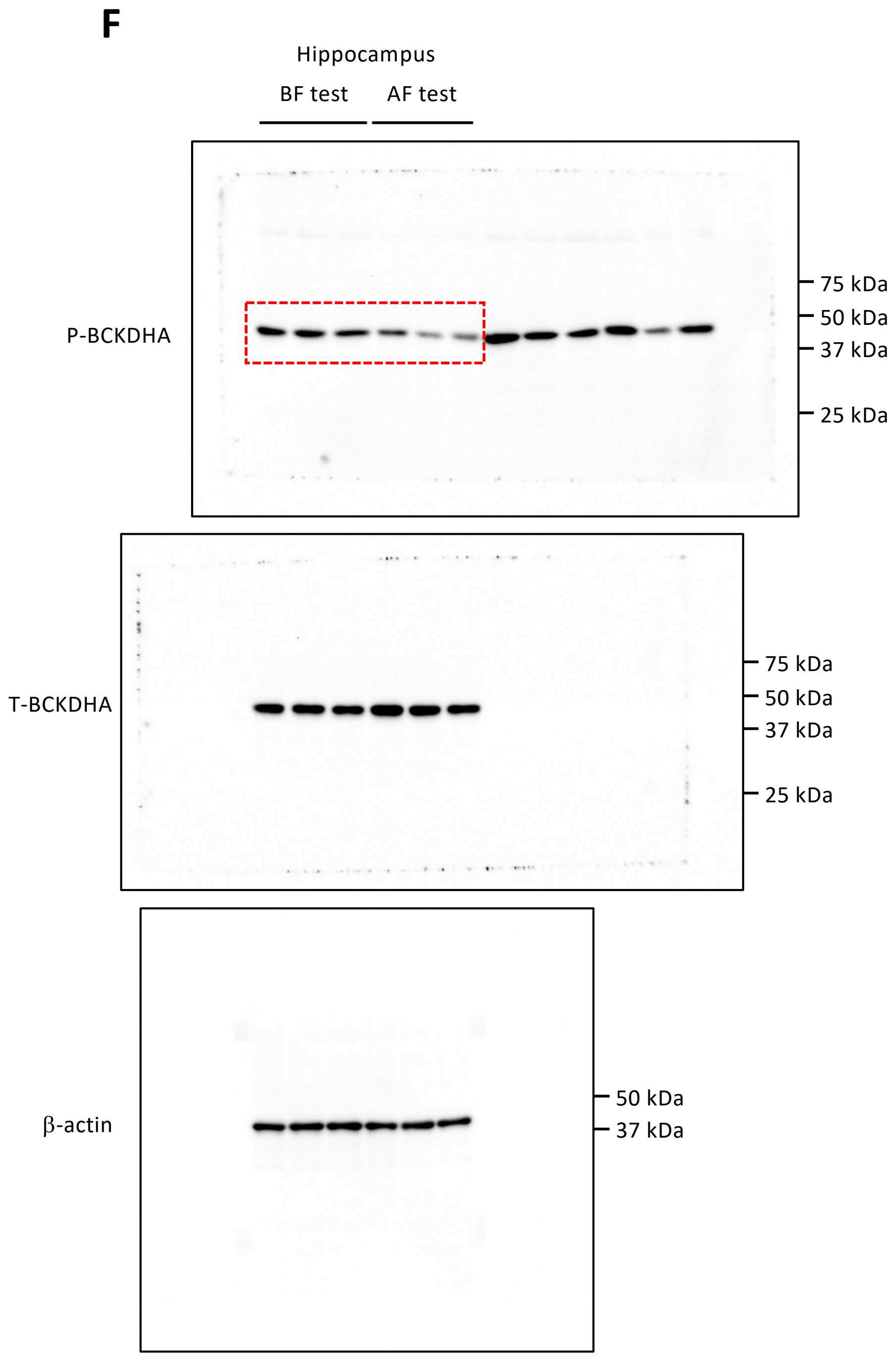

Supplement: Supplementary file 1 — Supplementary Figures. [file 41598_2021_2591_MOESM1_ESM.pdf]
